# Impact of a Research Integrity Assessment (RIA) of Randomized Controlled Trials Included in Interventional COVID‐19 Systematic Reviews: A Meta‐Epidemiological Study

**DOI:** 10.1002/cesm.70076

**Published:** 2026-03-15

**Authors:** Stephanie Weibel, Patrick Meybohm, Annika Oeser, Maria Popp, Tamara Pscheidl, Stefanie Reis, Lena Saal‐Bauernschubert, Stephanie Stangl, Emma Sydenham, Carina Wagner, Florencia Weber, Ana‐Mihaela Zorger, Nicole Skoetz

**Affiliations:** ^1^ Department of Anaesthesiology, Intensive Care, Emergency and Pain Medicine University Hospital Würzburg Würzburg Germany; ^2^ Institute of Public Health Faculty of Medicine and University Hospital Cologne, University of Cologne Cologne Germany; ^3^ Cochrane Central Editorial Service London UK

**Keywords:** evidence‐based medicine, meta‐analysis, meta‐epidemiological study, randomized controlled trials, research integrity, systematic reviews

## Abstract

**Objective:**

This study aimed to evaluate the feasibility, reliability, and impact of the Research Integrity Assessment (RIA) tool when applied to randomized controlled trials (RCTs) included in systematic reviews. RIA is a structured tool designed to assess retractions, trial registration, ethical approval, authorship, and plausibility of methods and results, thereby identifying RCTs that may not meet basic standards of research integrity.

**Design:**

Meta‐epidemiological study.

**Methods:**

We systematically identified Cochrane reviews and non‐Cochrane systematic reviews of RCTs investigating interventions for COVID‐19 and extracted all RCTs. Each RCT was independently assessed by two reviewers (with different expertise in evidence synthesis) using the RIA tool, with disagreements resolved by a senior reviewer. Reliability and feasibility were recorded, and sensitivity analyses examined the impact of excluding studies failing the RIA on meta‐analytic results.

**Results:**

Two hundred six RCTs from 23 Cochrane reviews and non‐Cochrane systematic reviews were assessed with RIA. Fifty‐nine RCTs (29%) were excluded due to integrity concerns, 79 (38%) classified as “awaiting classification”, and 11 (5%) identified as non‐randomized studies, leaving 57 RCTs (28%) rated as “no concern.” The most common reason for exclusion was absent or retrospective trial registration, while uncertainties around ethics approval were the main reason for “awaiting classification”. Interrater reliability was moderate overall (κ = 0.5), with higher agreement in objective domains and lower in domains requiring interpretive judgment, necessitating senior adjudication in a substantial proportion of assessments. On average, application of RIA required 21–27 min per RCT; however, the time required for senior assessor reassessment, conflict resolution, and author correspondence was not systematically recorded and substantially exceeded that of the initial assessments. We received 35 author responses to 165 individual queries. Sensitivity analyses restricted to RCTs passing RIA reduced the median number of eligible RCTs per meta‐analysis by 60%. This frequently widened confidence intervals and decreased the certainty of conclusions, although the direction of effect estimates changed only rarely.

**Conclusions:**

These results demonstrate that integrity checks can be feasibly applied and reveal widespread concerns in COVID‐19 RCTs, but require expertise, adjudication mechanisms, and adequate resources. Incorporating such assessments into review methodology can strengthen the trustworthiness of clinical evidence and safeguard patient safety.

**Registration:**

A protocol to this meta‐epidemiological study has been registered (https://doi.org/10.17605/OSF.IO/NBRHX).

## 1 Introduction

Evidence syntheses rely on the assumption that included studies adhere to good clinical practice and report valid results. However, problematic studies arising from misconduct, poor research practice, or honest error can undermine research integrity, distort systematic reviews and meta‐analyses, and ultimately threaten patient safety and human rights. False and fatally flawed studies have been found in the field of medicine [1, 2]. Retractions of such studies may influence reviews, guidelines, and clinical decision‐making, and findings would often change if such studies were excluded [3, 4, 5].

Beyond falsified or implausible data, non‐adherence to international standards for trial registration and ethical oversight also raises major concerns. The Declaration of Helsinki and the WHO mandate that clinical trials involving human participants must be prospectively registered in a publicly accessible database and approved by a research ethics committee before recruitment begins [6, 7]. Yet, most evidence syntheses do not address such violations or assess the risk of false or fabricated data. Therefore, transparent mechanisms to identify and manage problematic studies are essential to avoid misleading conclusions, safeguard adherence to international standards, and protect human rights.

In response, several groups have developed tools and checklists to assess the trustworthiness of clinical studies [4, 8, 9]. We contributed to these efforts by developing the Research Integrity Assessment (RIA), a structured tool to evaluate adherence of randomized controlled trials (RCTs) to principles of research integrity including compliance with good clinical practice [10]. It was first applied in the Cochrane review “Ivermectin for preventing and treating COVID‐19” where problematic studies were either excluded or placed in an “awaiting classification” category until clarification with the trial authors [10, 11]. Applying RIA led to the exclusion of over 40% of RCTs, most due to lack of prospective registration.

Building on this experience, the aim of the present study was to systematically apply RIA to a broader set of RCTs included in Cochrane reviews and non‐Cochrane systematic reviews of drug interventions for COVID‐19. Specifically, we sought to (1) test the feasibility and reliability of the RIA, (2) reanalyze meta‐analyses restricted to studies that passed the RIA, and (3) evaluate the impact of excluding problematic studies on review findings.

## 2 Methods

A protocol to this study has been published [12]. We followed the reporting guidelines for meta‐epidemiological research [13]. To identify the study pool, we used a systematic review approach to select systematic reviews of RCTs.

### 2.1 Inclusion Criteria for Systematic Reviews

Systematic reviews were identified based on predefined inclusion criteria. The pool of RCTs for the subsequent application of the RIA tool was derived from the primary studies included in the eligible systematic reviews.

#### 2.1.1 Study Design and Publication Format

We included systematic reviews that investigated RCTs of investigational medicinal products (IMPs [14, 15]) including investigational new drug applications (INDs [16, 17]) in humans, provided their search strategy, and defined their in‐ and exclusion criteria. We searched for Cochrane reviews and non‐Cochrane systematic reviews for each intervention of interest, with or without meta‐analysis. Pairwise and network meta‐analyses were eligible. We included full text, peer‐reviewed journal publications of systematic reviews. Preprints of systematic reviews, scoping reviews and narrative reviews were not eligible. We restricted the inclusion to publications in English.

#### 2.1.2 Study Population and Setting

We included systematic reviews investigating interventions for prevention or treatment of SARS‐CoV‐2 infection and COVID‐19 in humans, irrespective of SARS‐CoV‐2 diagnosis, disease severity or treatment setting.

#### 2.1.3 Interventions and Comparators

Relevant interventions for this study that have been investigated in systematic reviews were based on the topics published in the Cochrane COVID Review database (https://www.cochranelibrary.com/covid-19#Cochrane%20Reviews). As of June 8, 2022, 13 COVID‐19 Cochrane reviews investigating interventional drugs (IMPs/INDs) have been published, and were eligible for the current study: that is, antibiotics, anticoagulants, colchicine, convalescent plasma, hydroxychloroquine or chloroquine, inhaled corticosteroids, interleukin‐1 blocking agents, interleukin‐6 blocking agents, ivermectin, remdesivir, SARS‐CoV‐2‐neutralizing monoclonal antibodies, systemic corticosteroids, and vitamin D.

There were no restrictions regarding the comparator intervention. Any comparator was allowed, including no treatment, standard of care, placebo, or any active comparator.

### 2.2 Systematic Search

#### 2.2.1 Information Sources

We searched for Cochrane reviews and non‐Cochrane systematic reviews investigating the relevant interventions in Medline via PubMed to June 9, 2022. The search strategies can be found in File S1. We combined specific search strings for COVID‐19, systematic reviews and meta‐analyses (modified from Salvador‐Oliván [18]), and the relevant interventions.

#### 2.2.2 Study Selection

A systematic search followed by a two‐stage selection process was conducted to identify relevant studies.

In the systematic search, eligible Cochrane reviews and non‐Cochrane systematic reviews were identified through a comprehensive search in PubMed, focusing on study design, population, and relevant interventions. After deduplication using EndNote software, all records were independently screened by at least two systematic reviewers using Covidence (https://www.covidence.org/). Titles and abstracts were assessed for relevance; discrepancies were resolved through discussion or by involving a third systematic reviewer when necessary. Full‐text articles of potentially eligible systematic reviews were independently assessed by at least two systematic reviewers, with conflicts resolved by consensus or third‐party adjudication.

In the first selection stage (i.e., for systematic reviews), one systematic reviewer selected the Cochrane review (or update) and the non‐Cochrane systematic review (or update) for each of the 13 relevant interventions that included the largest pool of RCTs. The most recent publication date or the broadest inclusion criteria were prioritized. In the second selection stage (i.e., for RCTs), one systematic reviewer extracted all RCTs included in the selected systematic reviews, as well as those categorized as “awaiting classification” due to concerns regarding research integrity. Duplicates, non‐English RCTs, and RCTs available only as registered trial protocols, without reported results, were excluded. RCTs marked as “excluded” within the systematic reviews were not considered in the present analysis. The RIA tool was applied on the final pool of RCTs. We documented the screening and selection process of systematic reviews and RCTs in a PRISMA flow diagram including reasons for exclusion at the full‐text screening stage [19].

### 2.3 Data Collection Process and Data Items

Characteristics of eligible systematic reviews and included RCTs were extracted. One systematic reviewer extracted data of included Cochrane reviews and non‐Cochrane systematic reviews into a standardized MS Excel file. We examined how systematic reviews identified and addressed problematic studies, recording whether and how many were excluded or placed in an “awaiting classification“ category due to concerns about research integrity. One systematic reviewer retrieved all records of RCTs included in the systematic reviews, including full‐text publications, registered trial protocols and additional study documents (e.g., statistical analysis plan, supplementary data, trial protocols), which are necessary for the RIA, and extracted characteristics of the RCTs. All extractions made by the first systematic reviewer were checked by a second systematic reviewer.

### 2.4 RIA of RCTs Included in Systematic Reviews

RIA consists of six domains considering critical and important study criteria to assure research integrity of RCTs investigating IMPs based on adherence to good clinical practice and research integrity: retraction notices, prospective trial registration, ethics committee approval and written informed consent, study authorship, plausibility of methods, and plausibility of study results. The RIA tool, as well as its application, are described in detail elsewhere [10]. An Excel‐based format of the tool with critical and important signaling questions to the domains was used which is available online (https://doi.org/10.5281/zenodo.7024699). In an introductory online meeting, we explained the tool to all systematic reviewers (i.e., RIA assessors) and piloted its application on three studies.

The assessment of trial registration (RIA domain 2), including handling of inconsistencies, registry delays, and author correspondence, is described in detail in a separate meta‐epidemiological publication based on the same study pool [20]. The assessment of ethics approval and informed consent (RIA domain 3), including detailed extraction of ethics committee information, verification of committee recognition, author correspondence, and reliability considerations, has also been reported in depth in a separate publication based on the same study pool [21].

RIA was applied independently by two systematic reviewers. All RCTs were assessed by one reviewer (first assessor) who had little or no expertise in evidence synthesis (beginner; e.g., medical students or doctoral candidates with up to 1 year of experience). The second reviewer (second assessor) possessed a higher level of expertise, categorized as either intermediate (1–6 years of experience) or senior experts (> 6 years of experience) in evidence synthesis.

In the hierarchical workflow of RIA through domains 1–6, three decisions on a study's eligibility are possible at any step: RCTs may be either categorized as “no concern” (necessary for inclusion into a systematic review), “awaiting classification” or excluded. If the decision on one domain concluded that a RCT must be excluded, the following domains were omitted and were not assessed. After both assessors completed their assessments, the ratings of the first and second assessors were compared by a third assessor. Due to significant inconsistencies between assessors’ ratings, we decided post hoc to have a third assessor (senior expert in evidence synthesis) reassessing all RCTs in domains 1–3, as well as all RCTs in domains 4–6 that had been either classified as “awaiting classification“ or excluded by both assessors. Additionally, the third assessor re‐evaluated all RCTs in domains 4–6 where discrepancies existed between the two assessors’ ratings. This included RCTs that had been assessed by only one assessor, and those not yet assessed by any assessor due to prior exclusion decisions made in previous domains. We contacted the trial authors in case of inconsistency, insufficient information or serious concerns regarding any of the RIA domains. Each trial author had 2 weeks to respond and clarify the questions. Study authors who did not provide any feedback were reminded and given an additional 7 days to reply. If they did not respond or if questions remained unanswered, the RCT was moved to the “awaiting classification” category.

We recorded how often the first, second, and third assessors raised concerns about research integrity across the individual RIA domains and the overall RIA. We reported absolute and relative frequencies of RCTs that were classified as “no concern” (included), “awaiting classification”, or excluded.

### 2.5 Performance of RIA

Performance of RIA was assessed based on reliability and feasibility.

#### 2.5.1 Reliability of RIA

Reliability was assessed by measuring the interrater agreement between assessors on each domain, as well as on the overall RIA decision (derived from the cumulative domain assessments). Given the differing levels of expertise between the two assessors, agreement between a beginner and an intermediate/senior expert was examined. For each domain and the overall RIA decision, three possible ratings were defined: “no concern” (included in the overall RIA decision), “awaiting classification” and “excluded.” Interrater reliability was calculated for each domain based on the subset of RCTs that had been assessed independently by both assessors. We used the linear‐weighted Cohen's kappa (κ) statistic for three categories and interpreted κ as no agreement (κ < 0), slight (κ = 0.00–0.20), fair (κ = 0.21–0.40), moderate (κ = 0.41–0.60), substantial (κ = 0.61–0.80), or almost perfect agreement (κ = 0.81–1.00) [13]. We used the online calculator to quantify agreements (https://www.graphpad.com/quickcalcs/kappa1/).

#### 2.5.2 Feasibility of RIA

Feasibility was evaluated by recording the time required to apply the RIA tool to each domain, reported as the mean domain‐level assessment time across all assessors and all evaluated RCTs. Analyses distinguished between beginner and expert systematic reviewers. The total time taken to assess all domains was calculated as the sum of the mean times for each individual domain. Domains that were particularly time‐consuming or challenging to assess were identified. In addition, we recorded the number of author queries sent to clarify uncertainties regarding any RCT, as well as the proportion of responses received.

### 2.6 Impact of RIA on Systematic Reviews

To quantify the impact of the RIA on systematic reviews, we recorded the number of eligible RCTs per systematic review and per primary outcome meta‐analysis, both before and after applying the RIA. Across all systematic reviews, the median number of included RCTs was determined, along with the interquartile range (IQR). After applying the RIA, the number of RCTs rated as “no concern” was recorded, and the median proportion of originally included RCTs retained was calculated. In cases where systematic reviews combined RCTs and non‐randomized studies of interventions (NRSIs) in a meta‐analysis, only RCTs were considered. The same procedure was applied at the primary outcome level, assessing the median number of RCTs per meta‐analysis and the median proportion of RCTs remaining after the RIA.

We examined the impact of the RIA on meta‐analyses. Re‐analyses of meta‐analyses (i.e., sensitivity analyses) were performed for the primary outcome or for the first outcome of an outcome set of the systematic reviews excluding studies that failed the RIA. In case of different comparisons within a systematic review, we used the primary outcome of the comparison with the largest number of RCTs (or the largest number of participants, if the number of RCTs was equal) and estimable effects in the meta‐analysis. We only used patient‐relevant outcomes; laboratory values were not used for re‐analyses. We used the meta package version 8.2‐0 in R to perform meta‐analyses [22]. We employed the meta‐analysis methods outlined in the systematic reviews. In the sensitivity analyses, effect estimates from the original meta‐analyses were compared with those from re‐analyses excluding RCTs that failed the RIA according to three predefined dimensions: direction, precision, and interpretation.

- Direction was defined as the position of the point estimate relative to a prespecified equivalence range (RR/OR 0.9–1.11); a point estimate within the equivalence range indicates minimal or no effect; a point estimate < 0.9 indicates a potential benefit (e.g., for mortality) or a potential harm (e.g., for clinical improvement); a point estimate > 1.11 indicates a potential harm (e.g., for mortality) or a potential benefit (e.g., for clinical improvement).
- Precision was assessed based on whether the 95% confidence interval (CI) crossed one or more boundaries of this equivalence range; if the CI is entirely within one category (not crossing any boundary of the equivalence range), this indicates a precise effect; if the CI crosses at least one boundary of the equivalence range, this indicates an imprecise effect.
- Interpretation reflects the overall conclusion of the meta‐analysis and is derived from the combined assessment of the direction and precision of the effect estimate, resulting in either a precise benefit, harm, or no or minimal effect, or in uncertain effects (i.e., imprecise benefit, harm, or no or minimal effect).

We compared the proportion of meta‐analyses with changes in interpretation between Cochrane reviews and non‐Cochrane systematic reviews and reported the results descriptively. All analyses were summarized using absolute and relative frequencies of meta‐analyses showing changes in precision, direction, or overall interpretation.

For systematic reviews that used a risk of bias (RoB) assessment for RCTs included in meta‐analyses of the primary outcome, we descriptively compared the proportion of the overall RoB rating of RCTs that passed the RIA with that of RCTs that failed it.

### 2.7 Patient and Public Involvement

Patients and/or the public were not involved in the design, or conduct, or reporting, or dissemination plans of this research.

## 3 Results

### 3.1 Selection of Systematic Reviews and RCTs

**FIGURE 1:**
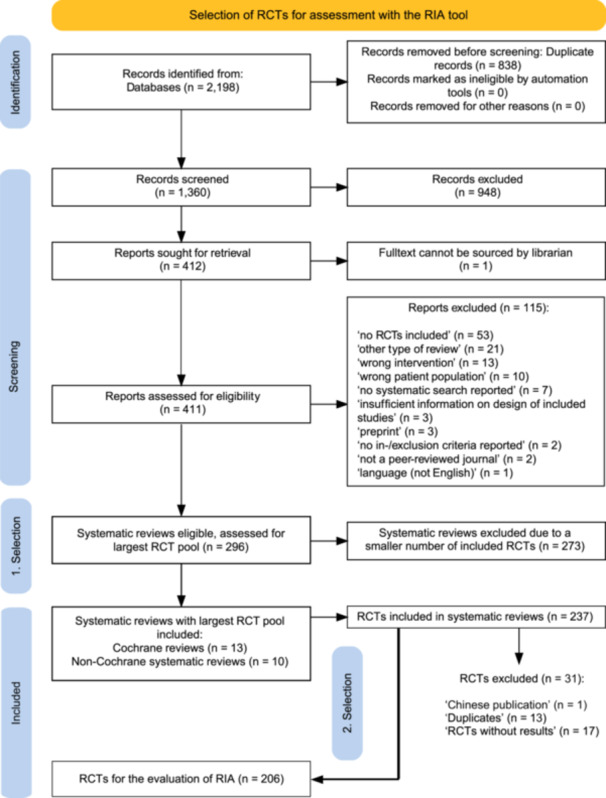
PRISMA flow diagram.

The search for Cochrane reviews and non‐Cochrane systematic reviews retrieved 2198 records and 838 duplicates were removed. Titles and abstracts of 1360 records were screened and 948 irrelevant records were excluded. One full‐text out of 412 reports sought for retrieval could not be found. Of 411 full‐text reports, 115 reports were excluded with reasons reported in Figure 1 and 296 systematic reviews were eligible. From these, 13 Cochrane reviews and 10 non‐Cochrane systematic reviews (three systematic reviews investigated two interventions each) with the largest pool of RCTs for each intervention were selected for this study, resulting in 23 systematic reviews encompassing 237 RCTs. Seven of these RCTs were classified at least in one systematic review as “awaiting classification” due to concerns with research integrity (File S2). We further identified and excluded 13 duplicate RCTs, 17 RCTs available only as registered trial protocols without reported results, and one non‐English‐language article, leaving 206 RCTs for the RIA. The selection process is illustrated in Figure 1.

#### 3.1.1 Characteristics of Systematic Reviews

Characteristics of the 13 Cochrane reviews [23, 24, 25, 26, 27, 28, 29, 30, 31, 32, 33, 34, 35] and the 10 non‐Cochrane systematic reviews [36, 37, 38, 39, 40, 41, 42, 43, 44, 45] are reported in File S2. Three non‐Cochrane systematic reviews providing the largest RCT pool performed network meta‐analysis, each covering two interventions: antibiotics and inhaled corticosteroids [45], convalescent plasma and SARS‐CoV‐2‐neutralizing monoclonal antibodies [37], and hydroxychloroquine or chloroquine and systemic corticosteroids [43]. Corresponding authors represented institutions in ten countries, most frequently Germany (39%, 9/23; all Cochrane reviews) and Canada (17%, 4/23; all non‐Cochrane systematic reviews). Funding was predominantly from government or research foundations (74%, 17/23); two systematic reviews reported no funding [36, 37], one was industry‐funded [38], and three did not report funding sources [39, 40, 42]. Most systematic reviews (78%, 18/23) included only RCTs, while five also included NRSIs, which were not eligible for RIA. The systematic reviews included between three [34] and 45 RCTs [43] for interventions of interest with a median number of 11 RCTs (interquartile range [IQR] 5–14). Eight Cochrane reviews held between three and 31 studies in the “awaiting classification” category; in four of these Cochrane reviews [29, 31, 32, 35], seven RCTs were flagged for concerns regarding research integrity, mainly due to implausible randomization or unclear methodology, and one due to implausible data (File S2). Four Cochrane reviews [26, 29, 30, 34] and one non‐Cochrane systematic review [39] excluded six studies for improper randomization (File S2). All‐cause mortality was the primary or first‐listed outcome in 87% (20/23) of systematic reviews. Two Cochrane reviews [24, 26] used clinical improvement as primary outcome, and one non‐Cochrane systematic review [42] used inflammatory laboratory markers. Risk of bias assessment was performed with the Cochrane RoB 2 tool at the outcome level in 74% (17/23) of systematic reviews; four used the RoB 1 tool at the study level, and two systematic reviews [36, 42] did not assess the risk of bias. One network meta‐analysis using RoB 2 did not report data for individual studies [43].

#### 3.1.2 Characteristics of RCTs

Characteristics and references of all 206 clinical studies assessed with RIA are provided in File S3. Of these, 11 were non‐randomized studies misclassified as RCTs in the systematic reviews: nine in Waheed et al. [36], one in Hosseini et al. [38], and one in Naveed et al. [42]. Among the 195 confirmed RCTs, 16% (32/195) were international multi‐center, 48% (93/195) national multi‐center, and 36% (70/195) single‐center studies. Of the 163 national multi‐center and single‐center RCTs, most were conducted in Asia (35%, 57/163), followed by Europe (25%, 41/163), South America (17%, 27/163), North America (15%, 24/163), and Africa (8%, 14/163). Frequent countries included the USA (18%, 17/93), UK (13%, 12/93), and Spain (9%, 8/93) for national multi‐center studies; and Iran (14%, 10/70), India (13%, 9/70), Brazil (10%, 7/70), and Egypt (10%, 7/70) for single‐center studies. The median number of randomized participants was 160 (IQR 78–506); 60 RCTs included ≤ 100 participants, predominantly from Asia (63%, 38/60). Regarding funding, most RCTs (47%, 91/195) were supported by public grants, followed by RCTs receiving full or partial industry funding (31%, 61/195), RCTs reporting no external funding (14%, 28/195), RCTs not reporting any funding source (8%, 15/195), and one RCT with private funding.

### 3.2 RIA Results—First Assessment Round

RIA was conducted independently by two assessors with differing levels of expertise in evidence synthesis. The first assessor (beginner) categorized the majority of RCTs as “awaiting classification” (51%, 105/206), excluded 45% (93/206), and rated 4% (8/206) as “no concern” (Table 1). Most exclusions (76%, 80/105) occurred in domain 2 (trial registration) due to missing prospective trial registration. The second assessor (intermediate/senior expert) classified most RCTs as “awaiting classification” (42%, 87/206), excluded 39% (80/206), and rated 19% (39/206) as “no concern” (Table 1), with most exclusions (64%, 51/80) also in domain 2 (trial registration). For both assessors, the predominant reason for assigning “awaiting classification” was uncertainty regarding domain 3 (ethics). Neither assessor excluded any RCT based on domain 6 (implausible study results).

**TABLE 1:** RIA by 1st assessor (beginner) and 2nd assessor (intermediate or senior expert); 206 RCTs.

Assessor's decision	Domain 1 (retraction)	Domain 2 (trial registration)	Domain 3 (ethics)	Domain 4 (authorship)	Domain 5 (plausibility of methods)	Domain 6 (plausibility of results)	Overall RIA
**1st assessor (beginner)**							
No. of RCTs assessed	206	199	119	115	115	113	206
No concern	190	76	33	102	63	48	8
Awaiting classification	9	43	82	13	50	65	105
Exclude	7	80	4	0	2	0	93
Not assessed^a^	0	7	87	91	91	93	0
Mean time needed/RCT	00:01:22	00:02:32	00:04:19	00:03:27	00:06:03	00:09:15	∑ 00:26:58
**2nd assessor (intermediate or senior expert)**							
No. of RCTs assessed	206	198	147	132	131	126	206
No concern	195	115	53	122	103	108	39
Awaiting classification	3	32	79	9	23	18	87
Exclude	8	51	15	1	5	0	80
Not assessed^a^	0	8	59	74	75	80	0
Mean time needed/RCT	00:01:05	00:02:03	00:03:49	00:03:20	00:03:36	00:06:59	∑ 00:20:51

Abbreviations: RCT, randomized controlled trial; RIA, research integrity assessment.

^a^ RCTs excluded in previous domains.

### 3.3 Reliability

Assessor comparisons revealed the highest numbers of discrepant ratings in domains 2, 3, 5, and 6 resulting in a discrepant rating of the overall RIA in 63 cases (Table 2). In domain 2 (trial registration), 46 RCTs were excluded by both assessors; however, experts rated RCTs more often as “no concern” than beginners, thus resulting in 74 discrepant ratings. In domain 3 (ethics), both assessors predominantly assigned “awaiting classification”, and produced 69 consistent and 45 discrepant ratings. In domains 5 and 6 (plausibility of methods and study results), beginners more frequently rated RCTs as “awaiting classification” whereas experts more often assigned “no concern”, yielding 36 and 50 discrepancies, respectively.

**TABLE 2:** Interrater agreement between 1st and 2nd assessors (first assessment round) across all RIA domains.

Interrater agreement	Domain 1 (retraction)	Domain 2 (trial registration)	Domain 3 (ethics)	Domain 4 (authorship)	Domain 5 (plausibility of methods)	Domain 6 (plausibility of results)	Overall RIA
No. of RCTs assessed by two assessors (1st and 2nd)	206	198	114	104	104	102	206
No. of RCTs with one rating^a^	0	1	38	39	38	35	0
No. of RCTs without rating^b^	0	7	54	63	64	69	0
TRUE (agreement, consistent rating)	196	124	69	96	68	52	143
FALSE (disagreement, discrepant rating)	10	74	45	8	36	50	63
% FALSE	4.85	37.37	39.47	7.69	34.61	49.02	30.58
κ	0.612	0.415	0.212	0.516	0.280	0.122	0.492
SE	0.108	0.048	0.085	0.148	0.080	0.054	0.048
Weighted κ	0.748	0.526	0.225	0.516	0.306	0.122	0.503
Interpretation	substantial	moderate	fair	moderate	fair	slight	moderate

Abbreviations: RCT, randomized controlled trial; RIA, research integrity assessment.

^a^ RCTs excluded in previous domains by one assessor.

^b^ RCTs excluded in previous domains by two assessors.

The current performance of the RIA tool showed limited interrater reliability. The hierarchical workflow, assessing domains 1–6 in sequence and omitting subsequent domains once a RCT was excluded, contributed substantially to variability between assessors. This structure led to a declining number of double‐assessed RCTs in later domains, from 206 in domain 1 to only 102 in domain 6 (Table 2). Agreement rates varied considerably across the domains (Table 2). Substantial agreement was observed in domain 1 (retraction; weighted κ = 0.748; 4.9% disagreement), while moderate agreement was observed in domain 2 (trial registration; κ = 0.526; 37.4% disagreement) and 4 (study authorship; κ = 0.516; 7.7% disagreement). Domains 3 (ethics) and 5 (plausibility of methods) showed only fair agreement (κ = 0.225 and κ = 0.306, respectively), while domain 6 (plausibility of study results) demonstrated slight agreement (κ = 0.122) with the highest proportion of disagreements (49.0%). The overall RIA agreement was moderate (κ = 0.503) with 30.6% disagreement.

### 3.4 RIA Results—3rd Assessor Reassessment

Due to the substantial discrepancies between first and second assessors, all RCTs meeting the criteria defined in the Methods section were re‐evaluated by a third assessor. The final RIA results for all 206 studies across all domains are provided in File S4 and is summarized in Table 3. Overall, 57 RCTs were classified as “no concern” 79 as “awaiting classification”, 59 were excluded, and 11 were identified as non‐randomized studies misclassified as RCTs in the systematic reviews. Exclusions were primarily due to absent or retrospective trial registration in 51 RCTs assessed in domain 2 (trial registration). Eight RCTs were excluded in domain 1 (retraction) due to retractions reported in the Retraction Watch Database [46]. No RCTs were excluded in domain 3–6. Misclassification as non‐randomized studies was detected in 10 cases during domain 2 (trial registration) assessment and in one case during domain 5 (plausibility of methods). The category “awaiting classification” was most often assigned in domain 3 for 62 RCTs due to uncertainties regarding ethics approval, followed by domains 2, 5, and 6, with 28 RCTs each.

**TABLE 3:** Third‐assessor reassessment and final RIA classification of RCTs.

Assessor's decision	Domain 1 (retraction)	Domain 2 (trial registration)	Domain 3 (ethics)	Domain 4 (authorship)	Domain 5 (plausibility of methods)	Domain 6 (plausibility of results)	Overall RIA
No. of RCTs	206	206	206	206	206	206	206
No. of RCTs assessed by 3rd assessor^a^/RCTs not excluded in previous domains	206/206	198/198	137/137	48/137	84/137	97/136^d^	NA
**No. of RCTs assessed by 1st and 2nd assessor for reassessment by 3rd assessor**							
with consistent rating	196	124	67	94	67	50	143
no concern	188	69	16	89	53	39	5
awaiting classification	1	9	49	5	13	11	69
exclude	7	46	2	0	1	0	69
with discrepant rating	10	74	45	8	35	49	63
with one rating	0	0	19	25	24	23	0
without rating	0	0	6	10	11	14	0
**No. of RCTs assessed by 3rd assessor**							
no concern	196	109	75	129	108	108	57
awaiting classification	2	28	62	8	28	28	79
exclude	8	51	0	0	0	0	59
identified as non‐RCT	0	10	0	0	1	0	11
not assessed^b^	0	8	69	69	69	70	0
No. of author requests sent	0	44	70^c^	2	24	25^e^	NA
No. of author responses received, and reclassified from “awaiting classification” to “no concern”	NA	11, 7	20, 17	0, 0	2, 1^d^	3, 3	NA

Abbreviations: RCT, randomized controlled trial; RIA, research integrity assessment.

^a^ A 3rd assessor assessed all RCTs for a domain which were not excluded or identified as non‐RCT in a previous domain following consensus. For domain 1, 2, and 3 all RCTs were assessed including those with consistent rating among 1st and 2nd assessor. For domain 4, 5, 6, RCTs with consistent rating were only assessed if rated “awaiting classification” or “exclude.”

^b^ RCTs excluded or identified as non‐RCT in previous domains.

^c^ One RCT did not report any contact email address in the primary trial report (CJWT629A12301); no author request sent.

^d^ one study turned out to be a non‐randomized study (Karakike‐2021).

^e^ Two contact email addresses were not available (Caricchio‐2021, Cremer‐2021).

### 3.5 Feasibility

Feasibility, measured as the time required to apply the RIA tool across all domains, averaged at approximately 27 min per RCT for beginners and 21 min for experts (Table 1). Across all assessors, domains 1 (retraction) and 2 (trial registration) required the least time, whereas domain 6 (plausibility of study results) was the most time‐consuming.

The time for third assessor reassessment, conflict resolution, and author correspondence was not recorded, although these steps were the most time‐consuming. Resolving a single “awaiting classification” case was estimated to take two to three times longer than the initial domain‐level assessment, mainly due to contacting trial authors. In total, 165 individual queries (potentially multiple per RCT) were sent to authors, yielding 35 responses; 28 RCTs were subsequently reclassified from “awaiting classification” to “no concern” after clarification (Table 3).

### 3.6 Impact of RIA on Systematic Reviews

The RIA reduced the number of eligible RCTs per systematic review from a median of 11 (5–14) to 3 (2–4) rated as “no concern” corresponding to a median of 32.8% (IQR 21.6%–47.9%) of those originally included (Table 4). At the primary outcome level, the number of eligible RCTs per (network) meta‐analysis decreased from a median of 7 (IQR 3–9) to 2 (IQR 1–4), representing a median of 40.4% (IQR 23.4%–66.7%) of the originally analyzed RCTs (Table 4). The impact of the RIA showed no difference in the median proportion of eligible RCTs that passed RIA between Cochrane reviews and non‐Cochrane systematic reviews (33.3% (IQR 21.4%–41.7%) vs. 32.3% (IQR 22.2%–50.0%)). However, a higher proportion of RCTs remained eligible at the meta‐analysis level in Cochrane reviews (50.0% (IQR 25.0%–71.4%)) compared to non‐Cochrane systematic reviews (33.3% (IQR 22.9%–40.7%)).

**TABLE 4:** Impact of RIA on systematic reviews: changes in direction, precision, and interpretation following sensitivity analyses.

Systematic review	Intervention of interest	No. RCTs included^a^/reports of RCTs RIA assessed	No. reports identified as non‐RCT	No. RCTs, RIA “no concern” (% of all included RCTs and RIA assessed reports)	Meta‐analysis of primary outcome^j^ reported in systematic review: No. RCTs, point estimate with 95% CI	Sensitivity analysis of primary outcome^j^: No. RCTs with RIA “no concern,” point estimate with 95% CI
Popp‐2021a	Antibiotics	11/11	0	6 (55%)	4, RR 0.98 (0.90 to 1.06)	3, RR 0.98 (0.90 to 1.06)
Zhang‐2021	Antibiotics	16/16	0	8 (50%)	12, NMA OR 0.94 (0.62 to 1.52)	8, not estimable
Flumignan‐2022	Anticoagulants	4/4	0	1 (25%)	4, RR 1.03 (0.92 to 1.16)	1, RR 1.00 (0.86 to 1.15)
Aamir Waheed‐2022	Anticoagulants	12/12	9	1 (8%)	9 (including 6 non‐RCTs), OR 0.63 (0.54 to 0.72)	1, OR 1.08 (0.85 to 1.38)
Mikolajewska‐2021	Colchicine	4/4	0	1 (25%)	2, RR 1.00 (0.93 to 1.08)	1, RR 1.01 (0.94 to 1.08)
Kow‐2022	Colchicine	10/10	0	3 (30%)	9, OR 0.76 (0.53 to 1.07)	3, OR 1.01 (0.93 to 1.10)
Piechotta‐2021	Convalescent plasma	12/12	0	5 (42%)	7, RR 0.98 (0.92 to 1.05)	5, RR 0.98 (0.92 to 1.05)
Deng‐2022	Convalescent plasma	32/32	0	13 (41%)	27, NMA OR 0.93 (0.84 to 1.03)	11, not estimable
Singh‐2021	Hydroxychloroquine or chloroquine	14/13^b^	0	5 (36%–38%)	9, RR 1.09 (0.99 to 1.19)	4^b^, RR 1.09 (0.99 to 1.19)
Siemieniuk‐2020	Hydroxychloroquine or chloroquine	45/31^c^	0	10 (22%–32%)	35, NMA RR 1.09 (0.93 to 1.27)	8^c^, not estimable
Griesel‐2022	Inhaled corticosteroids	3/3	0	2 (67%)	3, RR 0.61 (0.22 to 1.67)	2, RR 0.61 (0.22 to 1.67)
Zhang‐2021	Inhaled corticosteroids	4/4	0	2 (50%)	1, NMA OR 0.95 (0.63 to 1.69)	1, NMA OR 0.95 (0.63 to 1.69)
Davidson‐2022	Interleukin‐1 blocking agents	6/6	0	2 (33%)	3, RR 1.08 (0.97 to 1.20)	2, RR 1.12 (1.03 to 1.21)
Naveed‐2022	Interleukin‐1 blocking agents	4/4	1	1 (25%)	16 RCTs/NRSIs, RR 0.64 (0.49 to 0.83)	1 RCT, RR 0.93 (0.47 to 1.83)
					4 RCTs, RR 1.04 (0.76 to 1.41)^k^	
Ghosn‐2021	Interleukin‐6 blocking agents	10/10	0	2 (20%)	7, RR 1.06 (1.00 to 1.13)	1, RR 1.14 (1.08 to 1.21)
Yu‐2022	Interleukin‐6 blocking agents	17/16^d^	0	4 (24%–25%)	16, RR 0.88 (0.82 to 0.95)	4, RR 0.84 (0.78 to 0.91)
Popp‐2021b	Ivermectin	14/14	0	3^e^ (21%)	2, RR 0.60 (0.14 to 2.51)	0, not estimable
Izcovich‐2022	Ivermectin	29/29	0	4 (14%)	12, RR 0.50 (0.28 to 0.88)	1, RR 0.33 (0.01 to 8.05)
Ansems‐2021	Remdesivir	5/5	0	3 (60%)	4, RR 0.93 (0.81 to 1.06)	3, RR 0.90 (0.75 to 1.08)
Lee‐2022	Remdesivir	8/8	0	4 (50%)	8, RR 0.92 (0.78 to 1.08)	4, RR 0.91 (0.71 to 1.15)
Kreuzberger‐2021	SARS‐CoV‐2‐neutralizing monoclonal antibodies	6/10^f^	0	2^g^ (33%)	1, RR 0.94 (0.87 to 1.02)	1, RR 0.94 (0.87 to 1.02)
Deng‐2022	SARS‐CoV‐2‐neutralizing monoclonal antibodies	18/18	0	4 (22%)	5, NMA OR 0.86 (0.73 to 1.02)	2, not estimable
Wagner‐2021	Systemic corticosteroids	11/11	0	4 (36%)	9, RR 0.89 (0.80 to 1.00)	4, RR 0.90 (0.84 to 0.97)
Siemieniuk‐2020	Systemic corticosteroids	14/11^h^	0	4 (29%–36%)	11, NMA RR 0.83 (0.69 to 0.98)	4^h^, not estimable
Stroehlein‐2021	Vitamin D	3/3	0	0 (0%)	2, RR 0.58 (0.05 to 7.20)^l^	0, not estimable
Hosseini‐2022	Vitamin D	9/8^i^	1	0 (0%)	4 (RCT subgroup), RR 0.56 (0.25 to 1.25)	0, not estimable

*Note*: Color coding reflects the interpretation of the estimated effects based on direction and precision of the effect estimate. The range of equivalence was defined as 0.9 ≥ RR/OR ≤ 1.11, and the position of the point estimate relative to this range (direction) was assessed: yellow indicates a point estimate within the equivalence range (i.e., no or minimal effect); green indicates a point estimate < 0.9 (i.e., potential benefit for mortality and harm for clinical improvement); red indicates a point estimate > 1.11 (i.e., potential harm for mortality and benefit for clinical improvement). The 95% CI represents the precision of the estimate, and its relation to the equivalence range was also evaluated: dark green/yellow/red indicates a precise CI entirely within one category (not crossing any boundary of the equivalence range); light green/yellow/red indicates an imprecise CI crossing at least one boundary of the equivalence range.

Abbreviations: CI, confidence interval; NRSI, non‐randomized study of intervention; OR, odds ratio; RCT, randomized controlled trial; RIA, research integrity assessment tool; RR, risk ratio.

^a^ For interventions of interest of this meta‐epidemiological study.

^b^ One publication in Chinese language (Chen 2020)was excluded in this meta‐epidemiological study as the inclusion criteria was English language studies.

^c^ 13 RCTs without published results and one publication in Chinese language (Chen 2020) were excluded in this meta‐epidemiological study.

^d^ Two RCTs have been published in one article (17 studies); the publication (Hermine‐2022) was assessed only once with RIA.

^e^ The Cochrane review update (DOI: 10.1002/14651858.CD015017.pub3) did not exclude Pott‐Junior‐2021 due to concerns regarding the retraction of the publication.

^f^ 10 publications to six studies; study IDs according to registry record; different study phases and interim/full analyses counted as one study.

^g^ One study (NCT04425629) with three publications (Weinreich‐2021a, b, c) was assessed one time as “no concern” and two times as “awaiting classification.” We counted the study as “no concern.”

^h^ Three unpublished studies taken from a prospective meta‐analysis were excluded in this meta‐epidemiological study.

^i^ One RCT without results was excluded in this meta‐epidemiological study.

^j^ The primary outcome was all‐cause mortality at different time points except in three systematic reviews: the primary outcome was clinical improvement in Davidson‐2022 and Goshn‐2021, and COVID‐19‐related mortality in Hosseini‐2022.

^k^ Pooled effect estimates of RCTs only; the pooled estimate for RCTs only was not reported in the systematic review, which mixed RCTs and non‐RCTs in one meta‐analysis. The meta‐analysis of RCTs was recalculated by us using the method as described in the systematic review.

^l^ The Cochrane review did not report a pooled effect estimate; recalculated by us using the method as described in the systematic review.

Sensitivity analyses excluding all RCTs which were assessed as “awaiting classification” or excluded with RIA were conducted for meta‐analyses of primary patient‐relevant outcomes of 20 systematic reviews (Table 4). For three systematic reviews conducting network meta‐analyses, each for two interventions of interest, sensitivity analyses could not be conducted [37, 43, 45]. Of the 20 meta‐analyses, two reported precise effect estimates of benefit [36, 39], seven reported imprecise effect estimates of benefit [27, 31, 34, 35, 38, 40, 44], three reported precise effect estimates of no or minimal difference [29, 30, 32], and eight reported imprecise effect estimates of no or minimal difference [23, 24, 25, 26, 28, 33, 41, 42]. Sensitivity analyses did not change the interpretation of 11 effect estimates (Table 4). In the remaining nine effect estimates, sensitivity analyses mostly affected precision rather than direction: mortality outcomes had either no remaining RCTs in three meta‐analyses, or shifted from precise to imprecise effect estimates or from imprecise benefit to imprecise or no/minimal effect, with few changes in the overall direction (Table 4). For clinical improvement outcomes, effect estimates became slightly more favorable, changing from imprecise no or minimal effect to imprecise benefit (Table 4). In Cochrane reviews the interpretation changed in five out of 13 (38.5%) evaluated meta‐analyses [24, 26, 31, 34, 35]. In non‐Cochrane systematic reviews the interpretation changed in four out of seven (57.1%) evaluated meta‐analyses [36, 38, 39, 40]; six were network meta‐analyses, which were not subject to sensitivity analyses.

We compared the RoB assessments of RCTs that passed the RIA (“no concern”) with those that did not (excluded or “awaiting classification”) (Supporting File 5). Of 150 RCTs included in meta‐analyses with available RoB data, 61 passed the RIA and 89 did not; the distribution of RoB ratings was similar between the two RIA groups with low risk (37.7% vs. 30.3%), some concerns (39.3% vs. 48.3%), and high risk of bias (23.0% vs. 21.4%) indicating no relationship between RIA and RoB assessments.

## 4 Discussion

In this meta‐epidemiological study, systematic application of the RIA tool substantially reduced the number of RCTs retained in systematic reviews of COVID‐19 interventions. On median, only about one third of the originally included studies were classified as “no concern”. Given that only a small fraction of RCTs in our sample had been formally retracted, relying solely on retractions is insufficient to safeguard evidence syntheses. This finding highlights that potential issues related to research integrity and documentation are present in a considerable proportion of published RCTs, even within the highly regulated domain of clinical studies on SARS‐CoV‐2.

The reduction in eligible RCTs directly affected the precision of effect estimates in meta‐analyses. Sensitivity analyses showed that applying RIA occasionally shifted effect estimates towards no difference and more frequently widened confidence intervals, thereby reducing the certainty of conclusions. In the context of evidence‐based decision‐making, particularly for guideline development, such loss of precision and certainty can be critical, often resulting in more cautious or conditional recommendations.

A comparable impact was observed in the INSPECT‐SR Stage 2 study which tested a wide set of trustworthiness checks across RCTs from diverse Cochrane reviews [9]. There, applying integrity checks reduced the pool of eligible studies and frequently led to loss of precision in meta‐analyses, while rarely altering the overall direction or statistical significance of effects. Taken together, these findings across two independent approaches indicate that the primary impact of systematic integrity assessments is not a reversal of evidence signals but a reduction in certainty and precision. This reinforces the need for explicit integration of research integrity considerations into systematic review methodology, with the understanding that the resulting evidence base will often be smaller but more trustworthy.

Our findings add to the growing literature on methodological quality and integrity assessments, but extend it by implementing a structured, domain‐based approach at scale. While existing tools such as Cochrane's RoB 2 focus on internal validity, RIA addresses complementary dimensions, including prospective registration, ethics approval, and plausibility of methods and results. The absence of a relationship between RoB judgments and RIA classifications in our analysis and other studies underscores that these tools capture distinct methodological risks and should be viewed as complementary rather than interchangeable [9].

Across different applications of integrity screenings, prospective trial registration consistently emerges as a key indicator [4, 9, 47]. In our meta‐epidemiological study, absent or retrospective registration accounted for the majority of RCT exclusions. INSPECT‐SR similarly found that many RCTs were not prospectively registered and that such studies more often raised concerns regarding trial authenticity [9]. Importantly, lack of prospective registration should not be interpreted as evidence that a trial's results are false or fraudulent. Within the framework of the RIA, prospective registration is operationalised as a minimum integrity standard that enables verification of prespecified outcomes, recruitment timelines, study details, and protocol amendments. When registration is absent or retrospective, these elements cannot be reliably assessed, limiting confidence in transparency rather than demonstrating untrustworthiness per se. A comprehensive methodological description and in‐depth analysis of RIA domain 2 (trial registration), including its impact on eligibility decisions and assessor agreement, has been reported separately [20]. This meta‐epidemiological analysis of trial registration in the same study pool provides further context [20]. Prospectively registered RCTs were generally larger, longer, and more often multicentre, whereas non‐registered or retrospectively registered trials were more frequently small and single‐center. These associations indicate that prospective registration co‐occurs with established markers of trial design and organizational quality, although they do not establish a causal relationship with internal validity. This distinction is critical, as prospective registration serves a different role in integrity assessments than in conventional risk of bias evaluations, and whether it should justify exclusion or be addressed through sensitivity analyses remains an open empirical question.

Domain 3, which addresses ethics approval, was a frequent source of uncertainty in our assessments. In total, 62 RCTs were assigned to the “awaiting classification” category primarily due to unclear or missing information on ethics approval. A comprehensive methodological analysis of RIA domain 3 (ethics approval and informed consent), including reporting deficiencies, assessment reliability, and implications for integrity assessments in evidence synthesis, has been published separately and provides detailed context for the findings reported here [21]. This publication showed that inadequate reporting or documentation of ethics approval is common and can impede the evaluation of study trustworthiness [21]. The problem is further compounded by the fact that reporting of ethics approval is not yet a required CONSORT item for RCTs, limiting standardized guidance for authors and reviewers [48, 49]. These observations underscore the need for more explicit reporting standards to ensure that ethical oversight can be consistently documented as part of research integrity assessments.

No RCTs were excluded in domains 5 and 6 which assess plausibility of study methods and results, but 28 RCTs each were “awaiting classification” with unresolved concerns. In domain 5, trials with identical group sizes and insufficiently described randomization procedures were not automatically excluded; instead, authors were contacted to clarify allocation methods, and only unresolved cases were retained as “awaiting classification” The absence of exclusions in these domains therefore reflects a deliberately conservative exclusion threshold rather than a lack of identified concerns. One possible explanation is that objective tools or standardized procedures for testing methodological and data plausibility are currently lacking, making assessments in these domains largely dependent on subjective judgment and on the level of detail reported by trial authors. Implausible data patterns or inconsistencies are difficult to detect without access to individual participant data or advanced statistical forensics, which are rarely available in the context of systematic reviews. Where IPD are available, dedicated integrity tools (e.g., the IPD integrity tool) may support more reliable detection of anomalies than assessments based solely on aggregate reports [50]. While statistical forensics, such as those applied by Carlisle to detect anomalies in baseline data distributions, illustrate the potential to identify problematic trials, these approaches are not yet routinely implemented within systematic reviewing [51, 52]. As a result, potential issues in domains 5 and 6 may remain undetected, underscoring the need for more reliable and transparent approaches to evaluate plausibility of methods and results within research integrity assessments. Domain 6 should therefore be interpreted as a screening domain aimed at identifying obvious plausibility concerns rather than as a comprehensive forensic evaluation of numerical data.

A noteworthy finding is that seven Cochrane reviews and one non‐Cochrane systematic review in our sample had already applied research integrity considerations in their inclusion process without using a specific tool or checklist for their integrity assessments. Specifically, RCTs were excluded due to improper or implausible randomization, unclear methodology, or implausible results, while others placed studies with unresolved concerns into the “awaiting classification” category [26, 29, 30, 31, 32, 34, 35, 39]. Although the absolute numbers were small relative to the total body of evidence, these exclusions and reclassifications illustrate that integrity assessments are already influencing the evidence base of high‐quality systematic reviews. This underscores that even within Cochrane reviews, generally regarded as the benchmark for methodological rigor, integrity concerns can shape review conclusions, reinforcing the need for systematic and transparent procedures to evaluate research trustworthiness.

Reliability analysis indicated moderate interrater agreement overall (κ~0.5), with considerable variation between RIA domains. The lowest kappa values occurred in domains requiring substantial interpretive judgment (e.g., plausibility of study results), whereas domains with objective criteria (e.g., retraction) yielded higher agreement. The hierarchical structure of RIA may be one factor contributing to variability, as later domains were assessed in fewer studies, reducing the stability of reliability estimates for those domains. Differences between novice and experienced systematic reviewers suggest an important role for targeted training. The need for extensive third‐assessor involvement should be interpreted as an indicator of the complexity of integrity assessments rather than a failure of the tool, highlighting the importance of expertise and adjudication mechanisms for reliable application.

Regarding feasibility, RIA required on average 21–27 min per RCT in the first round of assessments. Cases rated as “awaiting classification” required two to three times longer in the second round of the assessments, mainly due to contacting trial authors and clarifying missing information or concerns. The authors response rate was low (~21%), underscoring the challenges of resolving integrity concerns. For routine application in systematic reviews, RIA would benefit from adequate resourcing or the development of more efficient screening strategies, potentially incorporating pre‐screening steps or semi‐automated support for objectively verifiable domains.

This study has several strengths, including the first large‐scale, standardized application of the RIA tool across a heterogeneous corpus of RCTs, inclusion of both Cochrane reviews and non‐Cochrane systematic reviews, and transparent documentation of all assessment decisions. Limitations include the post hoc decision to involve a third assessor for large parts of the assessment, which may have introduced systematic deviations from the protocol; the lack of systematic recording of time spent on conflict resolution; and restriction to English‐language reviews of COVID‐19 interventions, which may limit generalizability.

The implications for research and practice are substantial. For research, further validation and refinement of RIA are needed to improve both reliability and efficiency. Prospective studies should evaluate how RIA can be integrated into standard review workflows, and the potential for automation should be explored, particularly for domains with clear objective criteria. For practice, review teams should consider systematic integrity checks to strengthen the trustworthiness of evidence, particularly in fields with high publication volume or known integrity risks.

## 5 Conclusion

In conclusion, RIA identified substantial integrity concerns in a considerable share of RCTs and impacted the precision of the evidence base in systematic reviews. Implementing such assessments is feasible but requires expertise, resources, and standardized procedures. Further research should focus on broader applicability and on integrating integrity assessments into evidence‐based decision‐making processes.

## Author Contributions

Conceptualization: S.W., M.P., N.S., and E.S.; Data curation: S.W., T.P., and F.W.; Formal analysis: S.W., A.O., M.P., T.P., S.R., L.S.B., S.S., E.S., C.W., F.W., A.M.Z., and N.S.; Funding acquisition (departmental funding only): P.M. and N.S.; Investigation: S.W., T.P., and F.W.; Methodology: S.W., P.M., N.S., and E.S.; Project administration: S.W.; Supervision: S.W.; Writing – original draft: S.W.; Writing – review and editing: S.W., P.M., A.O., M.P., T.P., S.R., L.S.B., S.S., E.S., C.W., F.W., A.M.Z., and N.S.

## Ethics Statement

This study did not require ethics approval, as it involved the appraisal of already published research.

## Artificial Intelligence

ChatGPT (OpenAI) was used to support language editing; all final text was reviewed and approved by the authors.

## Conflicts of Interest

The authors declare no conflicts of interest. SW, PM, MP, NS, and CW authored Cochrane reviews included in this study. SW, ES, and FW contributed to the INSPECT‐SR project.

## Supporting information

Supplementary File 1 2025‐09‐24.

Supplementary File 2 2025‐09‐24.

Supplementary File 3 2025‐09‐24.

Supplementary File 4 2025‐09‐24.

Supplementary File 5 2025‐09‐24.

## Data Availability

All data relevant to this study are contained within the article, provided as supporting information, or available through an online repository linked with a digital object identifier (DOI).
