# Supplementary material for: Impact of a Research Integrity Assessment (RIA) of Randomized Controlled Trials Included in Interventional COVID‐19 Systematic Reviews: A Meta‐Epidemiological Study
Source: Cochrane Evid Synth Methods. 2026 Mar 15;4(2):e70076. doi: 10.1002/cesm.70076 (PMC13104099; doi:10.1002/cesm.70076)
Supplement: Supplementary file 1 — 2025‐09‐24. [file CESM-4-e70076-s005.docx]

**Supplementary File 1**: Search strategy for Cochrane reviews and non-Cochrane systematic reviews

**Search strategy (2022-06-09)**

**COVID-19 and systematic review/meta-analysis:**

#1 Search: (2019 nCoV[tiab] OR 2019nCoV[tiab] OR corona virus[tiab] OR corona viruses[tiab] OR coronavirus[tiab] OR coronaviruses[tiab] OR COVID[tiab] OR COVID19[tiab] OR nCov 2019[tiab] OR SARS-CoV2[tiab] OR SARS CoV-2[tiab] OR SARSCoV2[tiab] OR SARSCoV-2[tiab] OR "COVID-19"[Mesh] OR "COVID-19 Testing"[Mesh] OR "COVID-19 Vaccines"[Mesh] OR "Coronavirus"[Mesh:NoExp] OR "Receptors, Coronavirus"[Mesh] OR "SARS-CoV-2"[Mesh] OR "Spike Glycoprotein, Coronavirus"[Mesh]) NOT ("animals"[mh] NOT "humans"[mh]) NOT (editorial[pt] OR newspaper article[pt])

#2 Search: (systematic* [tiab] AND review [tiab]) OR Systematic overview* [tiab] OR Cochrane review* [tiab] OR systemic review* [tiab] OR scoping review [tiab] OR scoping literature review [tiab] OR mapping review [tiab] OR Umbrella review* [tiab] OR systematic review [pt] OR (review of reviews [tiab] OR overview of reviews [tiab]) OR meta-review [tiab] OR (integrative review [tiab] OR integrated review [tiab] OR integrative overview [tiab] OR meta- synthesis [tiab] OR metasynthesis [tiab] OR quantitative review [tiab] OR quantitative synthesis [tiab] OR research synthesis [tiab] OR meta-ethnography [tiab]) OR Systematic literature search [tiab] OR Systematic literature research [tiab] OR meta-analyses [tiab] OR metaanalyses [tiab] OR metaanalysis [tiab] OR meta-analysis [tiab] OR meta-analytic review [tiab] OR meta-analytical review [tiab] OR meta-analysis [pt] OR ((search* [tiab] OR medline [tiab] OR pubmed [tiab] OR embase [tiab] OR Cochrane [tiab] OR scopus [tiab] or web of science [tiab] OR sources of information [tiab] OR data sources [tiab] OR following databases [tiab]) AND (study selection [tiab] OR selection criteria [tiab] OR eligibility criteria [tiab] OR inclusion criteria [tiab] OR exclusion criteria [tiab]))

#3 Search: LETTER [PT] OR EDITORIAL [PT] OR COMMENT [PT] OR CASE REPORTS [PT] OR HISTORICAL ARTICLE [PT] OR REPORT [TI] OR PROTOCOL [TI] OR PROTOCOLS [TI]

#4 Search: #2 NOT #3

#5 Search: #1 AND #4

**Interventions:**

**Ivermectin** (source of search strategy: Popp M, Stegemann M, Metzendorf MI, Gould S, Kranke P, Meybohm P, et al. Ivermectin for preventing and treating COVID-19. Cochrane Database Syst Rev. Jul 28 2021;7:CD015017. doi:10.1002/14651858.CD015017.pub2)

#6 Search: ivermectin*[Title/Abstract] OR stromectol*[Title/Abstract] OR mectizan*[Title/Abstract] OR "MK 933"[Title/Abstract] OR MK933[Title/Abstract] OR eqvalan*[Title/Abstract] OR soolantra*[Title/Abstract] OR sklice*[Title/Abstract] OR stromectal*[Title/Abstract] OR ivomec*[Title/Abstract]

#7 Search: #5 AND #6

**Antibiotics** (source of search strategy: Popp M, Stegemann M, Riemer M, Metzendorf MI, Romero CS, Mikolajewska A, et al. Antibiotics for the treatment of COVID-19. Cochrane Database Syst Rev. Oct 22 2021;10(10):Cd015025. doi:10.1002/14651858.Cd015025)

#8 Search: antibio*[Title/Abstract] OR antimicrobi*[Title/Abstract] OR lactam*[Title/Abstract] OR monobactam*[Title/Abstract] OR penicillin*[Title/Abstract] OR Penizillin*[Title/Abstract] OR cephalospor*[Title/Abstract] OR macrolid*[Title/Abstract] OR tetrac*[Title/Abstract] OR Abramycin*[Title/Abstract] OR Abricycline[Title/Abstract] OR Achromycin[Title/Abstract] OR Agromicina[Title/Abstract] OR Ambramicina[Title/Abstract] OR Ambramycin[Title/Abstract] OR Amycin[Title/Abstract] OR "Bio‐tetra"[Title/Abstract] OR Biocycline[Title/Abstract] OR Cefracycline[Title/Abstract] OR Centet[Title/Abstract] OR Ciclibion[Title/Abstract] OR Copharlan[Title/Abstract] OR Criseociclina[Title/Abstract] OR Cyclomycin[Title/Abstract] OR Cyclopar[Title/Abstract] OR Democracin[Title/Abstract] OR Deschlorobiomycin[Title/Abstract] OR Hostacyclin[Title/Abstract] OR Lexacycline[Title/Abstract] OR Limecycline[Title/Abstract] OR Liquamycin[Title/Abstract] OR Mericycline[Title/Abstract] OR Micycline[Title/Abstract] OR Neocycline[Title/Abstract] OR Omegamycin*[Title/Abstract] OR Orlycyclin*[Title/Abstract] OR Panmycin[Title/Abstract] OR Piracaps[Title/Abstract] OR Polycyclin*[Title/Abstract] OR Polyotic[Title/Abstract] OR Purocyclina[Title/Abstract] OR Robitet[Title/Abstract] OR Roviciclina[Title/Abstract] OR Solvocin[Title/Abstract] OR Sumycin[Title/Abstract] OR Tetrabon[Title/Abstract] OR Tetradecin[Title/Abstract] OR Tetrafil[Title/Abstract] OR Tetraverin[Title/Abstract] OR Tsiklomistsin[Title/Abstract] OR Tsiklomitsin[Title/Abstract] OR Veracin[Title/Abstract] OR Vetacyclinum[Title/Abstract] OR Vetquamyc*[Title/Abstract] OR aminoglycosid*[Title/Abstract] OR lincosamid*[Title/Abstract] OR glycopeptid*[Title/Abstract] OR Amoxi*[Title/Abstract] OR Alfamox[Title/Abstract] OR Amodex[Title/Abstract] OR Amoksicillin*[Title/Abstract] OR Amophar[Title/Abstract] OR Amoran[Title/Abstract] OR Benzoral[Title/Abstract] OR Ciblor[Title/Abstract] OR Clamoxyl[Title/Abstract] OR Dispermox[Title/Abstract] OR Flemoxine[Title/Abstract] OR Galenamox[Title/Abstract] OR Gramidil[Title/Abstract] OR Hiconcil[Title/Abstract] OR Himinomax[Title/Abstract] OR Imacillin[Title/Abstract] OR Izoltil[Title/Abstract] OR Kentrocyllin[Title/Abstract] OR Larotid[Title/Abstract] OR Matasedrin[Title/Abstract] OR Metifarma[Title/Abstract] OR Moxal*[Title/Abstract] OR Novabritine[Title/Abstract] OR Pacetocin[Title/Abstract] OR Pamocil[Title/Abstract] OR Paradroxil[Title/Abstract] OR Polymox[Title/Abstract] OR Robamox[Title/Abstract] OR Siganopen[Title/Abstract] OR Simplamox[Title/Abstract] OR Sintopen[Title/Abstract] OR Trimox[Title/Abstract] OR Utimox[Title/Abstract] OR Velamox[Title/Abstract] OR Wymox[Title/Abstract] OR Zamocillin*[Title/Abstract] OR Zimox[Title/Abstract] OR clavulanic*[Title/Abstract] OR Clavulansäure[Title/Abstract] OR Ampicil*[Title/Abstract] OR Acillin[Title/Abstract] OR Adobacillin[Title/Abstract] OR Alpen[Title/Abstract] OR Amblosin[Title/Abstract] OR Amcill[Title/Abstract] OR Amfipen[Title/Abstract] OR "Amfipen V"[Title/Abstract] OR Aminobenzylpenicillin[Title/Abstract] OR "Amipenix S"[Title/Abstract] OR Ampi*[Title/Abstract] OR Amplacilina[Title/Abstract] OR Ampli*OR Ampy‐Penyl[Title/Abstract] OR Austrapen[Title/Abstract] OR Binotal[Title/Abstract] OR Bonapicillin[Title/Abstract] OR Britacil[Title/Abstract] OR Campicillin[Title/Abstract] OR Cimex[Title/Abstract] OR Copharcilin[Title/Abstract] OR "D‐Cillin"[Title/Abstract] OR Delcillin[Title/Abstract] OR Deripen[Title/Abstract] OR Divercillin[Title/Abstract] OR Doktacillin[Title/Abstract] OR Duphacillin[Title/Abstract] OR Grampenil[Title/Abstract] OR Guicitrina[Title/Abstract] OR Lifeampil[Title/Abstract] OR Morepen[Title/Abstract] OR Norobrittin[Title/Abstract] OR Nuvapen[Title/Abstract] OR "Olin Kid"[Title/Abstract] OR Omnipen[Title/Abstract] OR Orbicilina[Title/Abstract] OR "Pen Ampil"[Title/Abstract] OR Penbri*[Title/Abstract] OR Penbrock[Title/Abstract] OR Penicline[Title/Abstract] OR Penimic[Title/Abstract] OR Pensyn[Title/Abstract] OR Pentrex*[Title/Abstract] OR "Pfizerpen A"[Title/Abstract] OR Polycillin[Title/Abstract] OR Ponecil[Title/Abstract] OR Princillin[Title/Abstract] OR Principen[Title/Abstract] OR Qidamp[Title/Abstract] OR Sulbactam*[Title/Abstract] OR Piperacillin[Title/Abstract] OR Tazobactam*[Title/Abstract] OR Ceftriaxon*[Title/Abstract] OR Biotrakson[Title/Abstract] OR Rocephin[Title/Abstract] OR Cefotaxim*[Title/Abstract] OR Cephotaxim*[Title/Abstract] OR Claforan[Title/Abstract] OR Omnatax[Title/Abstract] OR Taxim[Title/Abstract] OR Clarithromycin*[Title/Abstract] OR Abbotic[Title/Abstract] OR Abbott[Title/Abstract] OR Adel[Title/Abstract] OR Astromen[Title/Abstract] OR Biaxin[Title/Abstract] OR Bicrolid[Title/Abstract] OR Clacine[Title/Abstract] OR Clambiotic[Title/Abstract] OR Claribid[Title/Abstract] OR Claricide[Title/Abstract] OR Clarith[Title/Abstract] OR Claritromicina[Title/Abstract] OR Clathromycin[Title/Abstract] OR Cyllid[Title/Abstract] OR Helas[Title/Abstract] OR Heliclar[Title/Abstract] OR Klacid[Title/Abstract] OR Klaciped[Title/Abstract] OR Klaricid[Title/Abstract] OR Klarid[Title/Abstract] OR Klarin[Title/Abstract] OR Klax[Title/Abstract] OR Kofron[Title/Abstract] OR Mabicrol[Title/Abstract] OR Macladin[Title/Abstract] OR Maclar[Title/Abstract] OR Mavid[Title/Abstract] OR Naxy[Title/Abstract] OR Veclam[Title/Abstract] OR Zeclar[Title/Abstract] OR Azithromycin*[Title/Abstract] OR Aritromicina[Title/Abstract] OR Azasite[Title/Abstract] OR Azenil[Title/Abstract] OR Azithromycin[Title/Abstract] OR Azitrocin[Title/Abstract] OR Azitromax[Title/Abstract] OR Azitromicin*[Title/Abstract] OR Aziwok[Title/Abstract] OR Aztrin[Title/Abstract] OR Hemomycin[Title/Abstract] OR Misultina[Title/Abstract] OR Mixoterin[Title/Abstract] OR Setron[Title/Abstract] OR Sumamed[Title/Abstract] OR Tobil[Title/Abstract] OR Tromix[Title/Abstract] OR Trulimax[Title/Abstract] OR "Z‐Pak"[Title/Abstract] OR Zeto[Title/Abstract] OR Zifin[Title/Abstract] OR Zithrax[Title/Abstract] OR Zithromax[Title/Abstract] OR Zitrim[Title/Abstract] OR Zitromax[Title/Abstract] OR Zitrotek[Title/Abstract] OR Zmas[Title/Abstract] OR Zmax[Title/Abstract] OR Doxycyclin*[Title/Abstract] OR Azudoxat[Title/Abstract] OR Deoxymykoin[Title/Abstract] OR Dossiciclina[Title/Abstract] OR Doxiciclina[Title/Abstract] OR Doxitard[Title/Abstract] OR Doxivetin[Title/Abstract] OR "Doxy‐Caps"[Title/Abstract] OR "Doxy‐Puren"[Title/Abstract] OR "Doxy‐Tabs"[Title/Abstract] OR Doxycen[Title/Abstract] OR Doxychel[Title/Abstract] OR Doxysol[Title/Abstract] OR Doxytetracycline[Title/Abstract] OR Investin[Title/Abstract] OR Liviatin[Title/Abstract] OR Monodox[Title/Abstract] OR Nordox[Title/Abstract] OR Oracea[Title/Abstract] OR Ronaxan[Title/Abstract] OR Spanor[Title/Abstract] OR "Vibra‐tabs"[Title/Abstract] OR Vibramycin*[Title/Abstract] OR Vibravenos[Title/Abstract] OR Zenavod[Title/Abstract] OR Moxifloxacin*[Title/Abstract] OR Avolex[Title/Abstract] OR Moxeza[Title/Abstract] OR Levofloxacin*[Title/Abstract] OR Ofloxacin[Title/Abstract] OR Cravit[Title/Abstract] OR Elequine[Title/Abstract] OR Iquix[Title/Abstract] OR Levaquin[Title/Abstract] OR Loxof[Title/Abstract] OR Oftaquix[Title/Abstract] OR Quixin[Title/Abstract] OR Tavanic[Title/Abstract] OR Unibiotic[Title/Abstract] OR Venaxan[Title/Abstract] OR Cefepim*[Title/Abstract] OR Maxipime[Title/Abstract] OR Ceftazidim*[Title/Abstract] OR Ceptaz[Title/Abstract] OR Fortaz[Title/Abstract] OR Pentacef[Title/Abstract] OR Tazicef[Title/Abstract] OR Tazidime[Title/Abstract] OR Imipenem*[Title/Abstract] OR Zienam[Title/Abstract] OR Cilastatin*[Title/Abstract] OR Meropenem*[Title/Abstract] OR Merrem[Title/Abstract] OR Ciprofloxacin[Title/Abstract] OR "Alcon Cilox"[Title/Abstract] OR AuriPro[Title/Abstract] OR Bacquinor[Title/Abstract] OR Baflox[Title/Abstract] OR Bernoflox[Title/Abstract] OR "Bi‐Cipro"[Title/Abstract] OR Cetraxal[Title/Abstract] OR Cifloxin[Title/Abstract] OR Cilab[Title/Abstract] OR Ciplus[Title/Abstract] OR Ciprecu[Title/Abstract] OR Ciriax[Title/Abstract] OR Citopcin[Title/Abstract] OR Cixan[Title/Abstract] OR Corsacin[Title/Abstract] OR Cycin[Title/Abstract] OR Cyprobay[Title/Abstract] OR Eni[Title/Abstract] OR Fimoflox[Title/Abstract] OR Ipiflox[Title/Abstract] OR Italnik[Title/Abstract] OR Linhaliq[Title/Abstract] OR Loxan[Title/Abstract] OR Otiprio[Title/Abstract] OR Probiox[Title/Abstract] OR Proflaxin[Title/Abstract] OR Proflox[Title/Abstract] OR Proksi[Title/Abstract] OR Quinolid[Title/Abstract] OR Quintor[Title/Abstract] OR Rancif[Title/Abstract] OR Roxytal[Title/Abstract] OR Septicide[Title/Abstract] OR "Sophixin Ofteno"[Title/Abstract] OR Spitacin[Title/Abstract] OR Superocin[Title/Abstract] OR Unex[Title/Abstract] OR Zumaflox[Title/Abstract] OR Gentamycin*[Title/Abstract] OR Centicin[Title/Abstract] OR Cidomycin[Title/Abstract] OR Garamycin[Title/Abstract] OR Garasol[Title/Abstract] OR Gentacycol[Title/Abstract] OR Gentalline[Title/Abstract] OR Gentamicin*[Title/Abstract] OR Gentavet[Title/Abstract] OR Gentocin[Title/Abstract] OR Lyramycin[Title/Abstract] OR Oksitselanim[Title/Abstract] OR Refobacin[Title/Abstract] OR "Refobacin TM"[Title/Abstract] OR Septigen[Title/Abstract] OR Uromycine[Title/Abstract] OR Tobra*[Title/Abstract] OR Epitobramycin[Title/Abstract] OR Aktob[Title/Abstract] OR Bethkis[Title/Abstract] OR "Deoxykanamycin B"[Title/Abstract] OR Gotabiotic[Title/Abstract] OR "Kitabis Pak"[Title/Abstract] OR "Lilly 47663"[Title/Abstract] OR Nebramycin[Title/Abstract] OR Tenebrimycin[Title/Abstract] OR Tenemycin[Title/Abstract] OR Tobacin[Title/Abstract] OR Tobi[Title/Abstract] OR Tobrex[Title/Abstract] OR Amikaci*[Title/Abstract] OR Amicaci*[Title/Abstract] OR Amiglyde[Title/Abstract] OR Amikavet[Title/Abstract] OR Amikozit[Title/Abstract] OR Amukin[Title/Abstract] OR Arikace[Title/Abstract] OR Kaminax[Title/Abstract] OR Lukadin[Title/Abstract] OR Mikavir[Title/Abstract] OR Pierami[Title/Abstract] OR Potentox[Title/Abstract] OR Metro*OR Acromona[Title/Abstract] OR Anagiardil[Title/Abstract] OR Arilin[Title/Abstract] OR Atrivyl[Title/Abstract] OR Bexon[Title/Abstract] OR Clont[Title/Abstract] OR CONT[Title/Abstract] OR Danizol[Title/Abstract] OR Deflamon[Title/Abstract] OR Efloran[Title/Abstract] OR Elyzol[Title/Abstract] OR Entizol[Title/Abstract] OR Eumin[Title/Abstract] OR Flagemona[Title/Abstract] OR Flagesol[Title/Abstract] OR Flagil[Title/Abstract] OR Flagyl[Title/Abstract] OR Fossyol[Title/Abstract] OR Giatricol[Title/Abstract] OR Gineflavir[Title/Abstract] OR Klion[Title/Abstract] OR Klont[Title/Abstract] OR Meronidal[Title/Abstract] OR Mexibol[Title/Abstract] OR Monagyl[Title/Abstract] OR Monasin[Title/Abstract] OR Nalox[Title/Abstract] OR "Neo‐tric"[Title/Abstract] OR NIDA[Title/Abstract] OR Noritate[Title/Abstract] OR Novonidazol[Title/Abstract] OR Orvagil[Title/Abstract] OR Protostat[Title/Abstract] OR Sanatrichom[Title/Abstract] OR Satric[Title/Abstract] OR Takimetol[Title/Abstract] OR Trichazol[Title/Abstract] OR Trichex[Title/Abstract] OR Tricho*[Title/Abstract] OR Tricocet[Title/Abstract] OR Tricom[Title/Abstract] OR "Tricowas B"[Title/Abstract] OR Trikacide[Title/Abstract] OR Trikamon[Title/Abstract] OR Trikojol[Title/Abstract] OR Trikozol[Title/Abstract] OR Trimeks[Title/Abstract] OR Trivazol[Title/Abstract] OR Vagilen[Title/Abstract] OR Vagimid[Title/Abstract] OR Vandazole[Title/Abstract] OR Vertisal[Title/Abstract] OR Wagitran[Title/Abstract] OR Lincomycin*[Title/Abstract] OR Cillimycin[Title/Abstract] OR Jiemycin[Title/Abstract] OR Lincolcina[Title/Abstract] OR Lincolnensin[Title/Abstract] OR Lincomicin*[Title/Abstract] OR Epilincomycin[Title/Abstract] OR Lincocin[Title/Abstract]

#9 Search: #5 AND #8

**Inhaled corticosteroids** (source of search strategy: Griesel M, Wagner C, Mikolajewska A, Stegemann M, Fichtner F, Metzendorf MI, et al. Inhaled corticosteroids for the treatment of COVID-19. Cochrane Database Syst Rev. Mar 9 2022;3(3):Cd015125. doi:10.1002/14651858.Cd015125)

#10 Search: corticosteroid*[Title/Abstract] OR corticoid*[Title/Abstract] OR prednison*[Title/Abstract] OR dehydrocortison*[Title/Abstract] OR deltason*[Title/Abstract] OR decortin*[Title/Abstract] OR orasone*[Title/Abstract] OR deltra*[Title/Abstract] OR meticorten*[Title/Abstract] OR cortancyl*[Title/Abstract] OR deltacorten*[Title/Abstract] OR dacortin*[Title/Abstract] OR adasone*[Title/Abstract] OR "delta‐cortison"[Title/Abstract] OR panasol*[Title/Abstract] OR decorton*[Title/Abstract] OR metacortandracin*[Title/Abstract] OR paracort*[Title/Abstract] OR predicor*[Title/Abstract] OR decortisyl*[Title/Abstract] OR delta‐1‐cortison*[Title/Abstract] OR "delta‐dome"[Title/Abstract] OR deltadehydrocortison*[Title/Abstract] OR ofisolon*[Title/Abstract] OR panafcort*[Title/Abstract] OR predicorten*[Title/Abstract] OR predni*[Title/Abstract] OR econonson*[Title/Abstract] OR promifen*[Title/Abstract] OR servison*[Title/Abstract] OR deltison*[Title/Abstract] OR lisacort*[Title/Abstract] OR meproson*[Title/Abstract] OR rayos[Title/Abstract] OR sterapred*[Title/Abstract] OR "liquid pred"[Title/Abstract] OR cortan*[Title/Abstract] OR rectodelt*[Title/Abstract] OR predeltin*[Title/Abstract] OR prednisolon*[Title/Abstract] OR methylprednisolon*[Title/Abstract] OR medrol[Title/Abstract] OR "pred forte"[Title/Abstract] OR medrone[Title/Abstract] OR urbason[Title/Abstract] OR wyacort[Title/Abstract] OR "Delta‐F"[Title/Abstract] OR duralon*[Title/Abstract] OR medrate[Title/Abstract] OR omnipred[Title/Abstract] OR adlone[Title/Abstract] OR caberdelta[Title/Abstract] OR depmedalon*[Title/Abstract] OR "Depo Moderin"[Title/Abstract] OR "Depo‐Nisolone"[Title/Abstract] OR Emmetipi[Title/Abstract] OR esameton*[Title/Abstract] OR firmacort[Title/Abstract] OR medlon*[Title/Abstract] OR "Mega‐Star"[Title/Abstract] OR meprolon*[Title/Abstract] OR metilbetason*[Title/Abstract] OR metrocort[Title/Abstract] OR metypresol[Title/Abstract] OR metysolon*[Title/Abstract] OR orapred[Title/Abstract] OR "Predni‐M‐Tablinen"[Title/Abstract] OR radilem[Title/Abstract] OR sieropresol[Title/Abstract] OR solpredon*[Title/Abstract] OR "A‐MethaPred"[Title/Abstract] OR prelone[Title/Abstract] OR aprednislon[Title/Abstract] OR pediapred[Title/Abstract] OR hostacortin[Title/Abstract] OR "Di‐Adreson‐F"[Title/Abstract] OR adnisolon*[Title/Abstract] OR capsoid[Title/Abstract] OR cortalon*[Title/Abstract] OR cortisolon*[Title/Abstract] OR deltacortril[Title/Abstract] OR estilsona[Title/Abstract] OR panafcortelone[Title/Abstract] OR sterane[Title/Abstract] OR "Delta‐Cortef"[Title/Abstract] OR econopred[Title/Abstract] OR dacortin[Title/Abstract] OR decaprednil[Title/Abstract] OR "Delta‐Diona"[Title/Abstract] OR "Delta‐Phoricol"[Title/Abstract] OR deltahydrocortison*[Title/Abstract] OR deltasolon*[Title/Abstract] OR deltidrosol[Title/Abstract] OR dhasolone[Title/Abstract] OR fisopred[Title/Abstract] OR frisolona[Title/Abstract] OR gupison*[Title/Abstract] OR hydeltra[Title/Abstract] OR hydeltrasol[Title/Abstract] OR klismacort[Title/Abstract] OR kuhlprednon[Title/Abstract] OR lenisolon*[Title/Abstract] OR "Lepi‐Cortinolo"[Title/Abstract] OR "Linola‐H"[Title/Abstract] OR longiprednil[Title/Abstract] OR metacortandralon*[Title/Abstract] OR "Meti Derm"[Title/Abstract] OR meticortelon*[Title/Abstract] OR opredsone[Title/Abstract] OR precortisyl[Title/Abstract] OR "Pred‐Clysma"[Title/Abstract] OR predeltilon*[Title/Abstract] OR prenilone[Title/Abstract] OR hydrocortancyl[Title/Abstract] OR "Solu Moderin"[Title/Abstract] OR predonin*[Title/Abstract] OR metypred[Title/Abstract] OR prednisol[Title/Abstract] OR dexamethason*[Title/Abstract] OR "BB 1101"[Title/Abstract] OR decadron[Title/Abstract] OR hexadrol[Title/Abstract] OR fortecortin[Title/Abstract] OR dexameth[Title/Abstract] OR dexone[Title/Abstract] OR hexadecadrol[Title/Abstract] OR desamethason*[Title/Abstract] OR ozurdex[Title/Abstract] OR deronil[Title/Abstract] OR baycuten[Title/Abstract] OR aacidexam[Title/Abstract] OR spersadex[Title/Abstract] OR dexacortal[Title/Abstract] OR gammacorten[Title/Abstract] OR visumetazon*[Title/Abstract] OR adexone[Title/Abstract] OR "Alba‐Dex"[Title/Abstract] OR cortidexason[Title/Abstract] OR decacort[Title/Abstract] OR decadrol[Title/Abstract] OR dectancyl[Title/Abstract] OR desameton[Title/Abstract] OR loverine[Title/Abstract] OR millicorten[Title/Abstract] OR orgadrone[Title/Abstract] OR alin[Title/Abstract] OR auxiloson[Title/Abstract] OR cortisumman[Title/Abstract] OR decalix[Title/Abstract] OR decameth[Title/Abstract] OR decasone[Title/Abstract] OR dekacort[Title/Abstract] OR deltafluorene[Title/Abstract] OR "Dexa‐Mamallet"[Title/Abstract] OR dexafluorene[Title/Abstract] OR dexalocal[Title/Abstract] OR dexamecortin[Title/Abstract] OR dexamonozon[Title/Abstract] OR dexapos[Title/Abstract] OR dexinoral[Title/Abstract] OR fluorodelta[Title/Abstract] OR lokalison[Title/Abstract] OR methylfluorprednisolon*[Title/Abstract] OR mymethason*[Title/Abstract] OR "Dexa‐Rhinosan"[Title/Abstract] OR "Dexa‐Scheroson"[Title/Abstract] OR "Dexa‐sine"[Title/Abstract] OR dexacortin[Title/Abstract] OR dexafarma[Title/Abstract] OR dinormon[Title/Abstract] OR baycadron[Title/Abstract] OR "Aeroseb‐Dex"[Title/Abstract] OR Maxidex[Title/Abstract] OR Dextenza[Title/Abstract] OR dexasone[Title/Abstract] OR dexpak[Title/Abstract] OR hydrocortison*[Title/Abstract] OR cortisol[Title/Abstract] OR cortef[Title/Abstract] OR hydrocorton*[Title/Abstract] OR cetacort[Title/Abstract] OR barseb[Title/Abstract] OR aeroseb[Title/Abstract] OR "Cort‐Dome"[Title/Abstract] OR cortenema[Title/Abstract] OR cortril[Title/Abstract] OR cortifan[Title/Abstract] OR cortispray[Title/Abstract] OR dermacort[Title/Abstract] OR domolene[Title/Abstract] OR eldecort[Title/Abstract] OR hautosone[Title/Abstract] OR "Heb‐Cort"[Title/Abstract] OR hytone[Title/Abstract] OR Komed[Title/Abstract] OR Nutracort[Title/Abstract] OR Proctocort[Title/Abstract] OR Rectoid[Title/Abstract] OR Hydrocort[Title/Abstract] OR locoid[Title/Abstract] OR Solu‐Glyc[Title/Abstract] OR glucocorticoid*[Title/Abstract] OR alclometason*[Title/Abstract] OR amcinonid*[Title/Abstract] OR beclomethason*[Title/Abstract] OR betamethason*[Title/Abstract] OR budesonid*[Title/Abstract] OR ciclesonid*[Title/Abstract] OR clobetas*[Title/Abstract] OR clocortolon*[Title/Abstract] OR desoximetason*[Title/Abstract] OR dichlorison*[Title/Abstract] OR diflorason*[Title/Abstract] OR diflucortolon*[Title/Abstract] OR difluprednate[Title/Abstract] OR drocinonid*[Title/Abstract] OR flumethason*[Title/Abstract] OR fluocinolon*[Title/Abstract] OR fluocinonid*[Title/Abstract] OR fluocortin[Title/Abstract] OR fluocortolon*[Title/Abstract] OR fluorometholon*[Title/Abstract] OR fluperolon*[Title/Abstract] OR flupredni*[Title/Abstract] OR flurandrenolone*[Title/Abstract] OR fluticason*[Title/Abstract] OR FX006[Title/Abstract] OR halometason*[Title/Abstract] OR medryson*[Title/Abstract] OR melengestrol[Title/Abstract] OR paramethason*[Title/Abstract] OR rimexolon*[Title/Abstract] OR terofenamat*[Title/Abstract] OR triamcinolon*[Title/Abstract] OR mometason*[Title/Abstract]

#11 Search: #5 AND #10

**Systemic corticosteroids** (source of search strategy: Wagner C, Griesel M, Mikolajewska A, Mueller A, Nothacker M, Kley K, et al. Systemic corticosteroids for the treatment of COVID-19. Cochrane Database Syst Rev. Aug 16 2021;8(8):Cd014963. doi:10.1002/14651858.Cd014963)

#12 Search: corticosteroid*[Title/Abstract] OR corticoid*[Title/Abstract] OR prednison*[Title/Abstract] OR dehydrocortison*[Title/Abstract] OR deltason*[Title/Abstract] OR decortin*[Title/Abstract] OR orasone*[Title/Abstract] OR deltra*[Title/Abstract] OR meticorten*[Title/Abstract] OR cortancyl*[Title/Abstract] OR deltacorten*[Title/Abstract] OR dacortin*[Title/Abstract] OR adasone*[Title/Abstract] OR "delta‐cortison"[Title/Abstract] OR panasol*[Title/Abstract] OR decorton*[Title/Abstract] OR metacortandracin*[Title/Abstract] OR paracort*[Title/Abstract] OR predicor*[Title/Abstract] OR decortisyl*[Title/Abstract] OR delta‐1‐cortison*[Title/Abstract] OR "delta‐dome"[Title/Abstract] OR deltadehydrocortison*[Title/Abstract] OR ofisolon*[Title/Abstract] OR panafcort*[Title/Abstract] OR predicorten*[Title/Abstract] OR predni*[Title/Abstract] OR econonson*[Title/Abstract] OR promifen*[Title/Abstract] OR servison*[Title/Abstract] OR deltison*[Title/Abstract] OR lisacort*[Title/Abstract] OR meproson*[Title/Abstract] OR rayos[Title/Abstract] OR sterapred*[Title/Abstract] OR "liquid pred"[Title/Abstract] OR cortan*[Title/Abstract] OR rectodelt*[Title/Abstract] OR predeltin*[Title/Abstract] OR prednisolon*[Title/Abstract] OR methylprednisolon*[Title/Abstract] OR medrol[Title/Abstract] OR "pred forte"[Title/Abstract] OR medrone[Title/Abstract] OR urbason[Title/Abstract] OR wyacort[Title/Abstract] OR "Delta‐F"[Title/Abstract] OR duralon*[Title/Abstract] OR medrate[Title/Abstract] OR omnipred[Title/Abstract] OR adlone[Title/Abstract] OR caberdelta[Title/Abstract] OR depmedalon*[Title/Abstract] OR "Depo Moderin"[Title/Abstract] OR "Depo‐Nisolone"[Title/Abstract] OR Emmetipi[Title/Abstract] OR esameton*[Title/Abstract] OR firmacort[Title/Abstract] OR medlon*[Title/Abstract] OR "Mega‐Star"[Title/Abstract] OR meprolon*[Title/Abstract] OR metilbetason*[Title/Abstract] OR metrocort[Title/Abstract] OR metypresol[Title/Abstract] OR metysolon*[Title/Abstract] OR orapred[Title/Abstract] OR "Predni‐M‐Tablinen"[Title/Abstract] OR radilem[Title/Abstract] OR sieropresol[Title/Abstract] OR solpredon*[Title/Abstract] OR "A‐MethaPred"[Title/Abstract] OR prelone[Title/Abstract] OR aprednislon[Title/Abstract] OR pediapred[Title/Abstract] OR hostacortin[Title/Abstract] OR "Di‐Adreson‐F"[Title/Abstract] OR adnisolon*[Title/Abstract] OR capsoid[Title/Abstract] OR cortalon*[Title/Abstract] OR cortisolon*[Title/Abstract] OR deltacortril[Title/Abstract] OR estilsona[Title/Abstract] OR panafcortelone[Title/Abstract] OR sterane[Title/Abstract] OR "Delta‐Cortef"[Title/Abstract] OR econopred[Title/Abstract] OR dacortin[Title/Abstract] OR decaprednil[Title/Abstract] OR "Delta‐Diona"[Title/Abstract] OR "Delta‐Phoricol"[Title/Abstract] OR deltahydrocortison*[Title/Abstract] OR deltasolon*[Title/Abstract] OR deltidrosol[Title/Abstract] OR dhasolone[Title/Abstract] OR fisopred[Title/Abstract] OR frisolona[Title/Abstract] OR gupison*[Title/Abstract] OR hydeltra[Title/Abstract] OR hydeltrasol[Title/Abstract] OR klismacort[Title/Abstract] OR kuhlprednon[Title/Abstract] OR lenisolon*[Title/Abstract] OR "Lepi‐Cortinolo"[Title/Abstract] OR "Linola‐H"[Title/Abstract] OR longiprednil[Title/Abstract] OR metacortandralon*[Title/Abstract] OR "Meti Derm"[Title/Abstract] OR meticortelon*[Title/Abstract] OR opredsone[Title/Abstract] OR precortisyl[Title/Abstract] OR "Pred‐Clysma"[Title/Abstract] OR predeltilon*[Title/Abstract] OR prenilone[Title/Abstract] OR hydrocortancyl[Title/Abstract] OR "Solu Moderin"[Title/Abstract] OR predonin*[Title/Abstract] OR metypred[Title/Abstract] OR prednisol[Title/Abstract] OR dexamethason*[Title/Abstract] OR "BB 1101"[Title/Abstract] OR decadron[Title/Abstract] OR hexadrol[Title/Abstract] OR fortecortin[Title/Abstract] OR dexameth[Title/Abstract] OR dexone[Title/Abstract] OR hexadecadrol[Title/Abstract] OR desamethason*[Title/Abstract] OR ozurdex[Title/Abstract] OR deronil[Title/Abstract] OR baycuten[Title/Abstract] OR aacidexam[Title/Abstract] OR spersadex[Title/Abstract] OR dexacortal[Title/Abstract] OR gammacorten[Title/Abstract] OR visumetazon*[Title/Abstract] OR adexone[Title/Abstract] OR "Alba‐Dex"[Title/Abstract] OR cortidexason[Title/Abstract] OR decacort[Title/Abstract] OR decadrol[Title/Abstract] OR dectancyl[Title/Abstract] OR desameton[Title/Abstract] OR loverine[Title/Abstract] OR millicorten[Title/Abstract] OR orgadrone[Title/Abstract] OR alin[Title/Abstract] OR auxiloson[Title/Abstract] OR cortisumman[Title/Abstract] OR decalix[Title/Abstract] OR decameth[Title/Abstract] OR decasone[Title/Abstract] OR dekacort[Title/Abstract] OR deltafluorene[Title/Abstract] OR "Dexa‐Mamallet"[Title/Abstract] OR dexafluorene[Title/Abstract] OR dexalocal[Title/Abstract] OR dexamecortin[Title/Abstract] OR dexamonozon[Title/Abstract] OR dexapos[Title/Abstract] OR dexinoral[Title/Abstract] OR fluorodelta[Title/Abstract] OR lokalison[Title/Abstract] OR methylfluorprednisolon*[Title/Abstract] OR mymethason*[Title/Abstract] OR "Dexa‐Rhinosan"[Title/Abstract] OR "Dexa‐Scheroson"[Title/Abstract] OR "Dexa‐sine"[Title/Abstract] OR dexacortin[Title/Abstract] OR dexafarma[Title/Abstract] OR dinormon[Title/Abstract] OR baycadron[Title/Abstract] OR "Aeroseb‐Dex"[Title/Abstract] OR Maxidex[Title/Abstract] OR Dextenza[Title/Abstract] OR dexasone[Title/Abstract] OR dexpak[Title/Abstract] OR hydrocortison*[Title/Abstract] OR cortisol[Title/Abstract] OR cortef[Title/Abstract] OR hydrocorton*[Title/Abstract] OR cetacort[Title/Abstract] OR barseb[Title/Abstract] OR aeroseb[Title/Abstract] OR "Cort‐Dome"[Title/Abstract] OR cortenema[Title/Abstract] OR cortril[Title/Abstract] OR cortifan[Title/Abstract] OR cortispray[Title/Abstract] OR dermacort[Title/Abstract] OR domolene[Title/Abstract] OR eldecort[Title/Abstract] OR hautosone[Title/Abstract] OR "Heb‐Cort"[Title/Abstract] OR hytone[Title/Abstract] OR Komed[Title/Abstract] OR Nutracort[Title/Abstract] OR Proctocort[Title/Abstract] OR Rectoid[Title/Abstract] OR Hydrocort[Title/Abstract] OR locoid[Title/Abstract] OR Solu‐Glyc[Title/Abstract]

#13 Search: #5 AND #12

**Anticoagulants** (source of search strategy, adapted: Reis S, Popp M, Schmid B, Stegemann M, Metzendorf MI, Kranke P, et al. Safety and Efficacy of Intermediate- and Therapeutic-Dose Anticoagulation for Hospitalised Patients with COVID-19: A Systematic Review and Meta-Analysis. J Clin Med. Dec 23 2021;11(1)doi:10.3390/jcm11010057)

#14 Search: anticoagula*[Title/Abstract] OR antithromb*[Title/Abstract] OR Thrombin Inhibitor*[Title/Abstract] OR Dabigatran[Title/Abstract] OR Pradaxa[Title/Abstract] OR Argatroban[Title/Abstract] OR Novastan[Title/Abstract] OR Acova[Title/Abstract] OR Lepirudin[Title/Abstract] OR Refludan[Title/Abstract] OR Desirudin[Title/Abstract] OR Iprivask[Title/Abstract] OR Revasc[Title/Abstract] OR desulfatohirudin*[Title/Abstract] OR recombinant HV1 hirudin[Title/Abstract] OR Bivalirudin[Title/Abstract] OR Hirulog*[Title/Abstract] OR Angiomax[Title/Abstract] OR Angiox[Title/Abstract] OR Xa inhibitor*[Title/Abstract] OR Xaban*[Title/Abstract] OR Rivaroxaban[Title/Abstract] OR Xarelto[Title/Abstract] OR Apixaban[Title/Abstract] OR Eliquis[Title/Abstract] OR Edoxaban[Title/Abstract] OR Lixiana[Title/Abstract] OR Savaysa[Title/Abstract] OR coumar*[Title/Abstract] OR cumar*[Title/Abstract] OR kumar*[Title/Abstract] OR Benzopyrone*[Title/Abstract] OR Benzopyran*[Title/Abstract] OR Hydroxycinnamic[Title/Abstract] OR Tonka bean camphor[Title/Abstract] OR Vitamin K antagonist[Title/Abstract] OR Vitamin K antagonists[Title/Abstract] OR phenprocoumon*[Title/Abstract] OR henylpropylhydroxycumarin*[Title/Abstract] OR Falithrom[Title/Abstract] OR Fencumar[Title/Abstract] OR Fenprocoumon*[Title/Abstract] OR Liquamar[Title/Abstract] OR Marcoumar[Title/Abstract] OR Marcumar[Title/Abstract] OR Phenprogramma[Title/Abstract] OR Warfarin*[Title/Abstract] OR Warfarat[Title/Abstract] OR Aldocumar[Title/Abstract] OR Warfant[Title/Abstract] OR Brumolin[Title/Abstract] OR Coumefene[Title/Abstract] OR Dethmor[Title/Abstract] OR Dethnel[Title/Abstract] OR Kypfarin[Title/Abstract] OR Marevan[Title/Abstract] OR Panwarfin[Title/Abstract] OR Prothromadin[Title/Abstract] OR Tedicumar[Title/Abstract] OR Zoocoumarin[Title/Abstract] OR Heparin*[Title/Abstract] OR Liquaemin[Title/Abstract] OR Adomiparin[Title/Abstract] OR Ardeparin[Title/Abstract] OR Arteven[Title/Abstract] OR Bemiparin*[Title/Abstract] OR Certoparin[Title/Abstract] OR Clexane[Title/Abstract] OR Klexane[Title/Abstract] OR Clivarin*[Title/Abstract] OR Dalteparin[Title/Abstract] OR Eparina[Title/Abstract] OR Fluxum[Title/Abstract] OR Fragmin A[Title/Abstract] OR Fragmin B[Title/Abstract] OR Fraxiparin[Title/Abstract] OR Hepathrom[Title/Abstract] OR Lipo- hepin[Title/Abstract] OR Liquemin[Title/Abstract] OR Multiparin[Title/Abstract] OR Nadroparin*[Title/Abstract] OR Novoheparin[Title/Abstract] OR Octaparin[Title/Abstract] OR Pabyrin[Title/Abstract] OR Parnaparin*[Title/Abstract] OR Parvoparin[Title/Abstract] OR Pularin[Title/Abstract] OR Reviparin[Title/Abstract] OR Sandoparin[Title/Abstract] OR Semuloparin[Title/Abstract] OR Subeparin[Title/Abstract] OR Sublingula[Title/Abstract] OR Thromboliquine[Title/Abstract] OR Tinzaparin*[Title/Abstract] OR Triofiban[Title/Abstract] OR Vetren[Title/Abstract] OR Vitrum AB[Title/Abstract] OR UFH[Title/Abstract] OR LMWH[Title/Abstract] OR Alphaparin*[Title/Abstract] OR Mono-Embolex[Title/Abstract] OR Enoxaparin*[Title/Abstract] OR Lovenox[Title/Abstract] OR Danaparoid[Title/Abstract] OR Danaproid[Title/Abstract] OR Orgaran[Title/Abstract] OR Lomoparan[Title/Abstract] OR Fondaparinux[Title/Abstract] OR Penta[Title/Abstract] OR Quixidar[Title/Abstract] OR Arixtra[Title/Abstract] OR sulodexid*[Title/Abstract] OR Aterina[Title/Abstract] OR Luzone[Title/Abstract] OR glucuronyl glucosamine glycan sulfate[Title/Abstract] OR glucuronyl glucosaminoglycan sulfate[Title/Abstract] OR Dociparastat[Title/Abstract]

#15 Search: #5 AND #14

**Anakinra** (source of search strategy: Kluge S. AWMF S3 Leitlinie – Empfehlungen zur stationären Therapie von Patienten mit COVID-19 – Living Guideline (Stand 02/2022). Accessed June 01, 2022. www.awmf.org/leitlinien/detail/ll/053-054.html)

#16 Search: Anakinra[Title/Abstract] OR IL1 Febrile Inhibitor[Title/Abstract] OR Interleukin 1 Inhibitor[Title/Abstract] OR Antril[Title/Abstract] OR Kineret[Title/Abstract] OR Interleukin 1 Receptor Antagonist[Title/Abstract] OR IL-1Ra[Title/Abstract] OR IL-1 Inhibitor[Title/Abstract]

#17 Search: #5 AND #16

**Cochicine** (source of search strategy: Mikolajewska A, Fischer AL, Piechotta V, Mueller A, Metzendorf MI, Becker M, et al. Colchicine for the treatment of COVID-19. Cochrane Database Syst Rev. Oct 18 2021;10(10):Cd015045. doi:10.1002/14651858.Cd015045)

#18 Search: colchicin*[Title/Abstract] OR colchicum[Title/Abstract] OR colchisol[Title/Abstract] OR colchysat[Title/Abstract] OR colcin[Title/Abstract] OR colcrys[Title/Abstract] OR colsaloid[Title/Abstract] OR condylon[Title/Abstract] OR goutnil[Title/Abstract] OR kolkicin[Title/Abstract] OR mitigare[Title/Abstract] OR demecolcin*[Title/Abstract] OR lumicolchicin*[Title/Abstract]

#19 Search: #5 AND #18

**mAbs** (source of search strategy: Kluge S. AWMF S3 Leitlinie – Empfehlungen zur stationären Therapie von Patienten mit COVID-19 – Living Guideline (Stand 02/2022). Accessed June 01, 2022. www.awmf.org/leitlinien/detail/ll/053-054.html)

#20 Search: ((((((antibod*[Title/Abstract] OR mAb[Title/Abstract] OR mAbs[Title/Abstract] OR nAb[Title/Abstract] OR nAbs[Title/Abstract]) AND (therap*[Title/Abstract] OR treat*[Title/Abstract] OR neutrali*[Title/Abstract]))) OR ((compet*[Title/Abstract] AND bind*[Title/Abstract]) OR (cocktail*[Title/Abstract] AND (mAb*[Title/Abstract] OR mAbs[Title/Abstract] OR antibod*[Title/Abstract] OR nAb*[Title/Abstract] OR nAbs[Title/Abstract])))) OR (((spike protein*[Title/Abstract] OR s protein*[Title/Abstract] OR Spike (S) protein[Title/Abstract])) OR (LY-3832479 OR LY3832479 OR LY-CoV016 OR REGN-COV2 OR REGN10933 OR REGN10987 OR casirivimab OR imdevimab OR LY-3819253 OR LY3819253 OR LY-CoV555 OR Bamlanivimab OR Banlanivimab OR VIR-7831 OR VIR7831 OR GSK4182136 OR GSK-4182136 OR sotrovimab OR AZD7442 OR AZD-7442 OR AZD1061 OR AZD-1061 OR AZD8895 OR AZD-8895 OR tixagevimab OR cilgavimab OR DXP593 OR DXP-593 OR BGB-DXP-593 OR BGBDXP593 OR JS016 OR JS-016 OR LY-CoV016 OR etesevimab OR TY027 OR TY-027 OR CTP59 OR CTP-59 OR CT-P59 OR regdanvimab OR STI1499 OR STI-1499 OR COVI-shield OR COVIshield OR COVI-guard OR COVIguard OR BRII196 OR BRII-196 OR SCTA01 OR SCTA-01 OR MW33 OR MW-33 OR BRII198 OR BRII-198 OR HFB30132A OR HFB-30132A OR ADM03820 OR ADM-03820 OR ADM03820 OR ADM-03820 OR HLX70 OR HLX-70 OR STI2020 OR STI-2020 OR COVIAMG OR COVI-AMG OR DZIF10c OR DZIF-10c OR BI767551 OR BI-767551 COV2-2381 OR COV22381 OR ABBV-47D11 OR 47D11 OR ABBV47D11 OR COR-101 OR COR101 OR STE90-C11 OR DXP-604 OR DXP604 OR BGB-DXP604 OR BGBDXP604 OR BGB-DXP-604 OR chicken egg antibod* OR anti-SARS-CoV-2 IgY* OR anti-SARS-CoV-2 IgYs OR egg yolk antibod* OR IgY OR IgYs)))) OR (spike protein, SARS-CoV-2[nm])

#21 Search: #5 AND #20

**Remdesivir** (source of search strategy: Ansems K, Grundeis F, Dahms K, Mikolajewska A, Thieme V, Piechotta V, et al. Remdesivir for the treatment of COVID-19. Cochrane Database Syst Rev. Aug 5 2021;8(8):Cd014962. doi:10.1002/14651858.Cd014962)

#22 Search: remdesivir* OR GS5734 OR GS 5734

#23 Search: #5 AND #22

**Vitamin D** (source of search strategy: Stroehlein JK, Wallqvist J, Iannizzi C, Mikolajewska A, Metzendorf MI, Benstoem C, et al. Vitamin D supplementation for the treatment of COVID-19: a living systematic review. Cochrane Database Syst Rev. May 24 2021;5(5):Cd015043. doi:10.1002/14651858.Cd015043)

#24 Search: vitamin d OR vitamind OR vitamin d3 OR vitamin d2 OR hydroxyvitamin d OR dihydroxyvitamin d OR cholecalciferol* OR colecalcifer* OR calciferol* OR calciol* OR calcidiol* OR calcitriol* OR calcifediol* OR calciferol* OR ercalcidiol* OR ercalcitriol* OR ergocalciferol* OR doxercalciferol* OR colecalciferol* OR paricalcitol* OR alphacalcidol* OR dihydrotachysterol*

#25 Search: #5 AND #24

**Convalescent plasma** (source of search strategy: Kluge S. AWMF S3 Leitlinie – Empfehlungen zur stationären Therapie von Patienten mit COVID-19 – Living Guideline (Stand 02/2022). Accessed June 01, 2022. www.awmf.org/leitlinien/detail/ll/053-054.html)

#26 Search: (((convalesc*[Title/Abstract] OR recovered[Title/Abstract] OR cured[Title/Abstract] OR rehabilitat*[Title/Abstract] OR survivor*[Title/Abstract] OR survived[Title/Abstract] OR virus-positive[Title/Abstract] OR virus neutrali*[Title/Abstract] OR virus inactivated[Title/Abstract] OR antibod*[Title/Abstract] OR high titre*[Title/Abstract] OR high titer*[All Fields]) AND (plasma[Title/Abstract] OR blood[Title/Abstract] OR donor*[Title/Abstract] OR donat*[Title/Abstract])) OR (therapeutic plasma[All Fields] OR plasma therapy[All Fields] OR immune plasma[All Fields] OR plasma exchange[All Fields] OR gamma globulin*[All Fields] OR gamma-Globulin[All Fields] OR hyper-Ig[All Fields]) OR (plasma[Title] AND (immun*[Title/Abstract] OR transfus*[Title/Abstract] OR infus*[Title/Abstract])) OR (high dos*[All Fields] AND (plasma[MeSH Terms] OR plasma[All Fields] OR plasmas[All Fields] OR plasma s[All Fields] OR immunoglobulin*[All Fields] OR ivig*[All Fields] OR ((immune[All Fields] OR immuned[All Fields] OR immunes[All Fields] OR immunisation[All Fields] OR vaccination[MeSH Terms] OR vaccination[All Fields] OR immunization[All Fields] OR immunization[MeSH Terms] OR immunisations[All Fields] OR immunizations[All Fields] OR immunise[All Fields] OR immunised[All Fields] OR immuniser[All Fields] OR immunisers[All Fields] OR immunising[All Fields] OR immunities[All Fields] OR immunity[MeSH Terms] OR immunity[All Fields] OR immunization s[All Fields] OR immunize[All Fields] OR immunized[All Fields] OR immunizer[All Fields] OR immunizers[All Fields] OR immunizes[All Fields] OR immunizing[All Fields]) AND globulin*[All Fields]) OR globulin*[All Fields])) OR (hyperimmune[All Fields] OR hyperimmunity[All Fields] OR hyperimmunization[All Fields] OR hyperimmunized[All Fields] OR hyperimmunizing[All Fields] OR hyper-immune[All Fields]) OR (serum[Title] OR sera[Title] OR serotherap*[Title/Abstract] OR sero therap*[Title/Abstract]) OR immunization, passive[MeSH Terms:noexp] OR (passiv*[Title/Abstract] AND ((antibod*[All Fields] AND transfer*[Title/Abstract]) OR immunisation*[Title/Abstract] OR immunization*[Title/Abstract] OR immunotherap*[Title/Abstract] OR immune therap*[Title/Abstract])) OR ((immunoglobulin*[Title] OR immune globulin*[Title]) AND (therap*[Title/Abstract] OR treat*[Title/Abstract]))) OR (equine*[Title/Abstract] OR hivig*[Title/Abstract]) OR (flu ivig*[Title/Abstract] OR ((anti flu*[Title/Abstract] OR anti influenza*[Title/Abstract] OR antiflu*[Title/Abstract] OR antinfluenza*[Title/Abstract]) AND plasma*[Title/Abstract])) OR COVID-19 serotherapy[Supplementary Concept]

#27 Search: #5 AND #26

**Tocilizumab**

#28 Search: ((((tocilizumab[Title/Abstract]) OR (anti-interleukin-6 receptor monoclonal antibody[Title/Abstract])) OR (Roactemra[Title/Abstract])) OR (Actemra[Title/Abstract])) OR (anti-IL-6 receptor monoclonal antibody[Title/Abstract])

#29 Search: #5 AND #28

**Hydroxychloroquine** (source of search strategy, adapted: Singh B, Ryan H, Kredo T, Chaplin M, Fletcher T. Chloroquine or hydroxychloroquine for prevention and treatment of COVID-19. Cochrane Database Syst Rev. Feb 12 2021;2(2):Cd013587. doi:10.1002/14651858.CD013587.pub2)

#30 Search: chloroquin*[Title/Abstract] OR Hydroxychloroquin*[Title/Abstract] OR Oxychloroquin*[Title/Abstract] OR Aralen[Title/Abstract] OR Plaquenil[Title/Abstract] OR antimalaria*[Title/Abstract] OR anti‐malaria*[Title/Abstract]

#31 Search: #5 AND #30
