# Supplementary material for: Impact of a Research Integrity Assessment (RIA) of Randomized Controlled Trials Included in Interventional COVID‐19 Systematic Reviews: A Meta‐Epidemiological Study
Source: Cochrane Evid Synth Methods. 2026 Mar 15;4(2):e70076. doi: 10.1002/cesm.70076 (PMC13104099; doi:10.1002/cesm.70076)
Supplement: Supplementary file 5 — 2025‐09‐24. [file CESM-4-e70076-s003.docx]

**Supplementary File 5**

| **Systematic review (RoB tool used)** | **Intervention of interest** | **Primary outcome^1^;**  **No. RCTs** | **Primary outcome** | | **Primary outcome** | |
| --- | --- | --- | --- | --- | --- | --- |
|  |  |  | **No. RCTs with RIA ‘no concern’** | **Overall^2^ RoB for RCTs with RIA ‘no concern’** | **No. RCTs with RIA ‘exclude’ plus ‘awaiting classification’** | **Overall^2^ RoB for RCTs with RIA ‘exclude’ or ‘awaiting classification’** |
| Popp-2021a  (RoB 2) | Antibiotics | 4 | 3 | Low: 100%  Some concerns: 0%  High: 0% | 1 | Low: 0%  Some concerns: 100%  High: 0% |
| Zhang-2021  (RoB 2) | Antibiotics | 12 | 8 | Low: 12.5%  Some concerns: 0%  High: 87.5% | 4 | Low: 50%  Some concerns: 0%  High: 50% |
| Flumignan-2022  (RoB 1) | Anticoagulants | 4 | 1 | Low: 0%  Some concerns: 0%  High: 100% | 3 | Low: 0%  Some concerns: 0%  High: 100% |
| Aamir Waheed-2022  (without RoB assessment) | Anticoagulants | 9 (6 non-RCTs) | 1 | Low: NA  Some concerns: NA  High: NA | 2 (6 non-RCTs) | Low: NA  Some concerns: NA  High: NA |
| Mikolajewska-2021  (RoB 2) | Colchicine | 2 | 1 | Low: 0%  Some concerns: 0%  High: 100% | 1 | Low:0 %  Some concerns: 0%  High: 100% |
| Kow-2022  (RoB 2) | Colchicine | 9 | 3 | Low: 0%  Some concerns: 100%  High: 0% | 6 | Low: 0%  Some concerns: 83.3%  High: 16.6% |
| Piechotta-2021  (RoB 2) | Convalescent plasma | 7 | 5 | Low: 40%  Some concerns: 60%  High: 0% | 2 | Low: 100%  Some concerns: 0%  High: 0% |
| Deng-2022  (RoB 2) | Convalescent plasma | 27 | 11 | Low: 18.2%  Some concerns: 72.7%  High: 9.1% | 16 | Low: 6.3%  Some concerns: 75%  High: 18.7% |
| Singh-2021  (RoB 1) | Hydroxychloroquine or chloroquine | 9^3^ | 4 | Low: 25%  Some concerns: 25%  High: 50% | 4 | Low: 0%  Some concerns: 0%  High: 100% |
| Siemieniuk-2020  (RoB 2) | Hydroxychloroquine or chloroquine | 35^4^ | 8 | Low: NR  Some concerns: NR  High: NR | 13 | Low: NR  Some concerns: NR  High: NR |
| Griesel-2022  (RoB 2) | Inhaled corticosteroids | 3 | 2 | Low: 50%  Some concerns: 50%  High: 0% | 1 | Low: 100%  Some concerns: 0%  High: 0% |
| Zhang-2021  (RoB 2) | Inhaled corticosteroids | 1 | 1 | Low: 0%  Some concerns: 0%  High: 100% | 0 | Low: NA  Some concerns: NA  High: NA |
| Davidson-2022  (RoB 2) | Interleukin-1 blocking agents | 3 | 2 | Low: 50%  Some concerns: 50%  High: 0% | 1 | Low: 0%  Some concerns: 100%  High: 0% |
| Naveed-2022  (without RoB assessment) | Interleukin-1 blocking agents | 4 | 1 | Low: NA  Some concerns: NA  High: NA | 2 (1 non-RCT) | Low: NA  Some concerns: NA  High: NA |
| Ghosn-2021  (RoB 2) | Interleukin-6 blocking agents | 7 | 1 | Low: 0%  Some concerns: 100%  High: 0% | 6 | Low: 50%  Some concerns: 50%  High: 0% |
| Yu-2022  (RoB 1) | Interleukin-6 blocking agents | 16 | 4 | Low: 75.0%  Some concerns: 25%  High: 0% | 12 | Low: 50%  Some concerns: 50%  High: 0% |
| Popp-2021b  (RoB 2) | Ivermectin | 2 | 0 | Low: NA  Some concerns: NA  High: NA | 2 | Low: 0%  Some concerns: 100%  High: 0% |
| Izcovich-2022  (RoB 2) | Ivermectin | 12 | 1 | Low: 100%  Some concerns: 0%  High: 0% | 11 | Low: 45.5%  Some concerns: 18.2%  High: 36.3% |
| Ansems-2021  (RoB 2) | Remdesivir | 4 | 3 | Low: 100%  Some concerns: 0%  High: 0% | 1 | Low: 0%  Some concerns:100 %  High: 0% |
| Lee-2022  (RoB 2) | Remdesivir | 8 | 4 | Low: 100%  Some concerns: 0%  High: 0% | 4 | Low: 50%  Some concerns: 0%  High: 50% |
| Kreuzberger-2021  (RoB 2) | SARS-CoV-2-neutralising monoclonal antibodies | 1 | 1 | Low: 0%  Some concerns: 0%  High: 100% | 0 | Low: NA  Some concerns: NA  High: NA |
| Deng-2022  (RoB 2) | SARS-CoV-2-neutralising monoclonal antibodies | 5 | 2 | Low: 50%  Some concerns: 50%  High: 0% | 3 | Low: 33.3%  Some concerns: 66.7%  High: 0% |
| Wagner-2021  (RoB 2) | Systemic corticosteroids | 9 | 4 | Low: 0%  Some concerns: 100%  High: 0% | 5 | Low: 20%  Some concerns: 80%  High: 0% |
| Siemieniuk-2020  (RoB 2) | Systemic corticosteroids | 11^5^ | 4 | Low: NR  Some concerns: NR  High: NR |  | Low: NR  Some concerns: NR  High: NR |
| Stroehlein-2021  (RoB 2) | Vitamin D | 2 | 0 | Low: NA  Some concerns: NA  High: NA | 2 | Low: 50%  Some concerns: 50%  High: 0% |
| Hosseini-2022  (RoB 1) | Vitamin D | 4 | 0 | Low: NA  Some concerns: NA  High: NA | 4 | Low: 50%  Some concerns: 50%  High: 0% |

Abbreviations: non-randomised study of intervention (NRSI); randomized controlled trial (RCT); research integrity assessment tool (RIA); risk of bias (RoB)

Footnotes

^1^ the primary outcome was all-cause mortality at different time points except in three systematic reviews: the primary outcome was clinical improvement in Davidson-2022 and Goshn-2021, and COVID-19-reated mortality in Hosseini-2022

^2^ We used the overall RoB per RCT as reported by the systematic review if RoB 2 was used; we assumed the overall RoB based on the results of the individual domains if RoB 1 was used and an overall RoB was not reported in the systematic review

^3^ one publication in Chinese language was excluded in this meta-epidemiological study and not assessed with RIA

^4^ 13 RCTs without published results and one publication in Chinese language were excluded in this meta-epidemiological study and not assessed with RIA

^5^ 3 RCTs without published results were excluded in this meta-epidemiological study and not assessed with RIA
